# Wavenumber Calibration Protocol for Raman Spectrometers Using Physical Modelling and a Fast Search Algorithm

**DOI:** 10.1177/00037028241254847

**Published:** 2024-06-02

**Authors:** Dongyue Liu, Bryan M. Hennelly

**Affiliations:** 1 Department of Electronic Engineering, 8798 Maynooth University, Kildare, Ireland; 2 Department of Computer Science, 8798 Maynooth University, Kildare, Ireland

**Keywords:** Wavenumber calibration, Czerny–Turner spectrograph, transmission spectrometer, reference wavenumber standard

## Abstract

A wavenumber calibration protocol is proposed that replaces polynomial fitting to relate the detector axis and the wavenumber shift. The physical model of the Raman spectrometer is used to derive a mathematical expression relating the detector plane to the wavenumber shift, in terms of the system parameters including the spectrograph focal length, the grating angle, and the laser wavelength; the model is general to both reflection and transmission gratings. A fast search algorithm detects the set of parameters that best explains the position of spectral lines recorded on the detector for a known reference standard. Using three different reference standards, four different systems, and hundreds of spectra recorded with a rotating grating, we demonstrate the superior accuracy of the technique, especially in bands outside of the outermost reference peaks when compared with polynomial fitting. We also provide a thorough review of wavenumber calibration for Raman spectroscopy and we introduce several new evaluation metrics to this field borrowed from chemometrics, including leave-one-out and leave-half-out cross-validation.

## Graphical abstract

**Graphical abstract:**
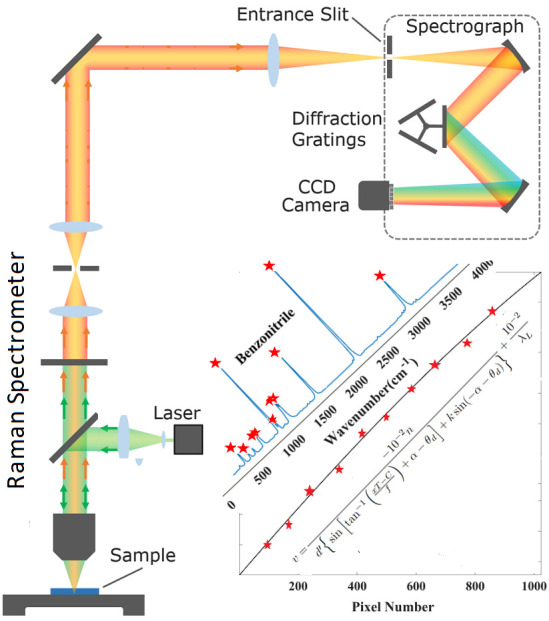
This is a visual representation of the abstract.

## Introduction

The objective of this paper is to develop a direct wavenumber calibration protocol for Raman spectroscopy (RS) that outperforms traditional methods. The concept is to build upon the conventional protocol of applying low-order polynomial fitting to the spectral peak positions of a wavenumber standard such as 4-acetamidophenol. We replace the typical polynomial fitting step with the fitting of a nonlinear function that represents the model of the optical system which is the spectrometer. This function is governed by the various parameters that describe the optical system such as the grating period and focal length and it is necessary to search over these parameters in order to find an optimal fitting to the available peak positions. Although the laser wavelength is one of the parameters, we believe the approach can be described as a “direct” protocol, because only an approximate value of the wavelength is required as input.

Raman spectroscopy (RS) probes the vibrational and rotational modes of molecules whereby laser photons scattered by the material have lost energy related to the energy of certain Raman-active molecular bonds present within the sample. RS can identify biomolecular changes within cells as they progress from a healthy to a cancerous state^1–3^ making it a powerful technique for the identification of cancer cells and tissue. Post-processing such as multivariate statistical analysis^4,5^ is typically applied to Raman spectra for classification, whereby statistical pattern recognition algorithms identify subtle changes across data sets that can be used to accurately differentiate between different pathological groups.^1–3,6–11^ One application of particular commercial interest is Raman guided surgery.^12–14^ Another emerging area of clinical research is automated Raman cytology.^15–19^

The development of the clinical application of RS is hindered by poor cross-instrument comparability, which has been highlighted by two recent multi-site studies,^20,21^ both of which demonstrate inconsistencies in the wavenumber shift for various materials even following established calibration protocols provided by the instrument manufacturer. Itoh et al.^21^ examined spectra from polystyrene, benzonitrile, and cyclohexane obtained across 26 different systems from which they concluded that the wavenumber shift inconsistencies resulted from the instrumentation and calibration protocols and not from the materials samples. Guo et al.^20^ found similar inconsistencies across 35 different instruments using acetaminophen, polystyrene, and cyclohexane. Cross-instrument differences relate to both wavenumber shift as well as intensity variation for the same sample, the latter being caused by the differing wavelength and polarization-dependent transmission function of each instrument and possibly differing instrument resolution as well as other potential causes. Wavenumber variation is generally attributed to small changes in the instrument resulting from thermal expansion or positional drift; instruments with motorized gratings are particularly susceptible to miscalibration. These studies have highlighted the need for further research into the cause of cross-instrument variability and for the availability of open-access standardized materials and calibration protocols that can be universally adopted.

There has already been some development of consensus standards for Raman instrumentation by the American Society for Testing and Materials (ASTM) international in relation to performance testing, calibration, and relative intensity correction (ASTM E1683,^22^ E1840,^23^ E2529,^24^ and E2911^25^). An excellent review of these standards and the various calibration protocols are provided in the literature.^26–28^ Further information on reference materials certified by the Chinese and Japanese metrology institutes provides an expanded uncertainty over those by ASTM.^28^ In summary, these standards relate to methods for spectral response correction and wavenumber calibration for a single instrument, and for evaluating the performance of the instrument in terms of resolution, stray light, sensitivity, etc. In the context of this paper, the most relevant of these standards is ASTM-E1840,^23^ most recently updated in 2022, which focuses on Raman shift calibration. Included in this document are the Raman shift values for eight wavenumber standards including acetaminophen and benzonitrile, which we utilize in this study; these values were determined by eight independent laboratories and only the most stable peaks, standard deviation (SD) $<1\;{\mathrm{cm}}^{-1}$ , were included. Interestingly, this guide does not set out a particular method of calibration; two approaches are commonly used in the literature: (i) wavelength calibration using an atomic spectrum such as from neon followed by wavenumber conversion making use of the laser wavelength, and (ii) direct use of a Raman wavenumber standard such as acetaminophen. In the Supplemental Information, we review both of these approaches in some detail and we conclude that direct wavenumber standards are preferable as they do not require knowledge of the laser wavelength and can provide accuracy and precision at least as good as wavelength calibration.

All of the literature to date, on the subject of direct wavenumber calibration employs a low-order polynomial (typically of order 3) to fit the detector pixel and reference wavenumber shift pairs that are recorded from a reference standard; this polynomial provides the calibrated wavenumber shift axis. In this paper, we propose an alternative to polynomial fitting, which provides superior accuracy and precision, particularly in bands outside of the outermost peaks in the wavenumber reference spectrum. The method is based on deriving the relationship between wavenumber shift and detector pixel for an arbitrary Raman spectrometer based on the physical model, which is defined in terms of the system parameters including the spectrometer focal length, grating angle, etc. A search algorithm estimates the set of parameters that are optimal in terms of fitting the detector pixel and reference wavenumber shift pairs. The method is tested on hundreds of spectra recorded using four different systems with varying resolutions including a reflection Czerny–Turner spectrometer with a motorized grating as well as a low f -number spectrometer with a holographic transmission grating. In all cases, it is shown that the method is superior to polynomial fitting and we believe that this method could be considered for inclusion in future iterations of ASTM-E1840.^23^ We recognize that we are not the first to introduce a direct wavenumber calibration protocol based on a physical model. It is common for commercial Raman instruments to come with manufacturer-provided calibration procedures tailored to the specific spectrometer. Some of these procedures may incorporate a physical model of the spectrometer as part of the direct wavenumber calibration process using a Raman reference. However, to the best of our knowledge, none of these protocols have been published or made publicly available.

It is important to acknowledge that the wavenumber calibration protocol proposed in this paper is directly related to our recently published paper that proposed a wavelength calibration protocol using much the same approach.^29^ In that paper, the equation that describes optical propagation within the spectrometer was used to replace the third-order polynomial that is conventionally applied to fit to the peak positions in a neon spectrum. In this paper, we build on this in a simple manner by applying the wavenumber conversion formula to the aforementioned equation, providing a new equation that relates the Raman wavenumber shift to the detector pixel. This latter equation is then used to fit to the peaks of a wavenumber reference standard such as 4-acetamidophenol. The difference between the calibration routines in this paper and in Hennelly and Liu^29^ is, therefore, only slight; the fitting equation, which although different in form, has only one extra parameter in the laser wavelength. Secondly, the reference spectra are Raman spectra of wavenumber reference standards instead of atomic emission wavelength reference lamps.

The breakdown of this paper is as follows: A physical model of the general Raman spectrometer is analyzed in the following section, and the relationship between wavenumber shift and detector pixel is derived; we also investigate what polynomial order would best fit this relationship, which has been a subject of debate in previous papers. Based on this relationship, an algorithm is proposed to replace polynomial fitting. A methods section provides a detailed step-by-step description of the overall calibration routine and the metrics used for accuracy/precision. Finally, results are presented, which are followed by a brief conclusion. We note that a Supplemental Information is provided that includes a comprehensive background section in which a thorough review of wavenumber calibration is provided that includes a comparison of the two approaches mentioned above. This Supplemental Information document also provides additional results and clarification that are referenced throughout the paper.

## Relationship Between Wavenumber Shift and Pixel Position in a Spectrometer

### Relationship Between Wavelength and Pixel Position for a Generalized Spectrometer with a Rotating Grating

**Figure 1.**
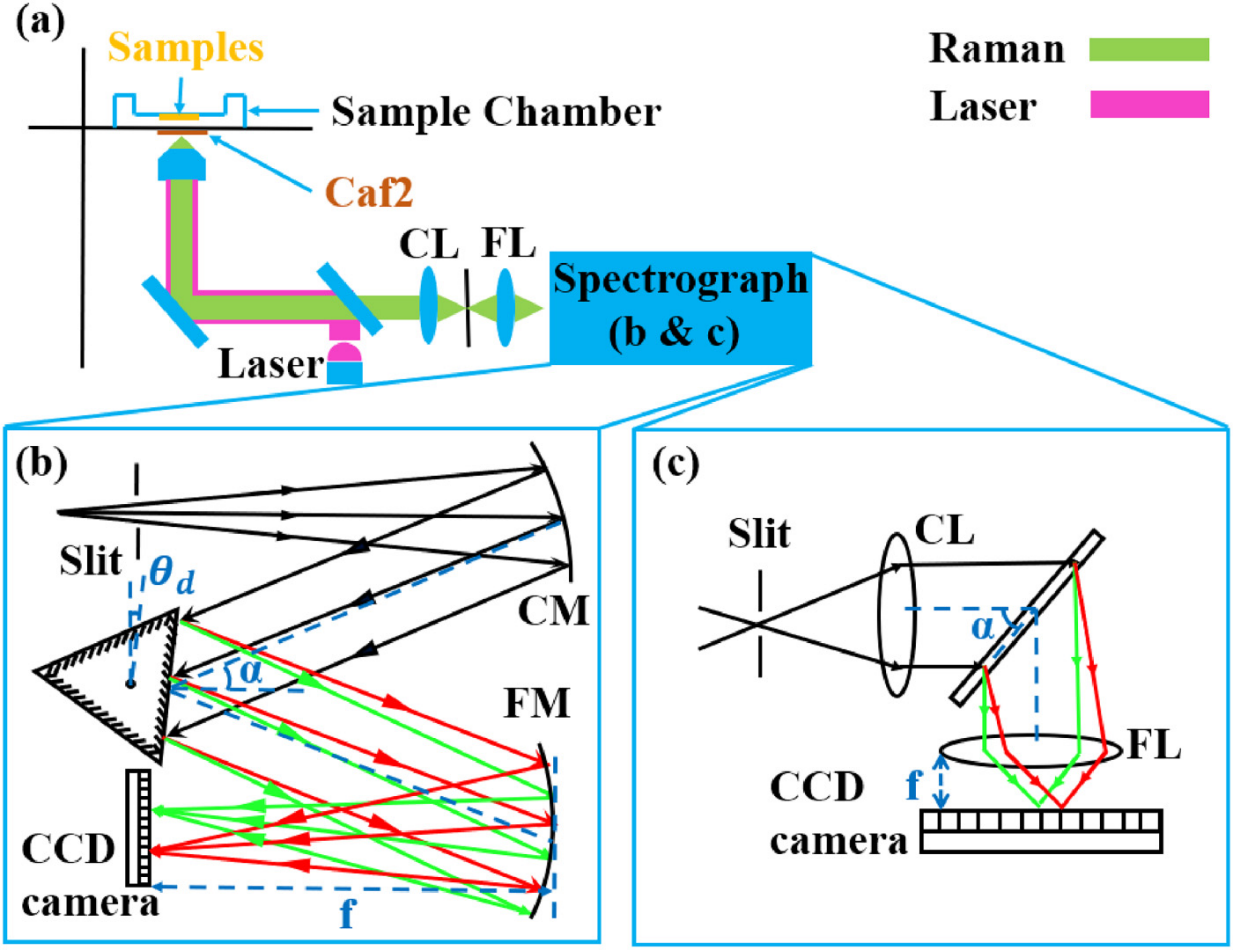
(a) Basic Raman spectrometer with epi-illumination; (b) A Czerny–Turner spectrograph with a rotating grating; the parameters shown in the illustration appear in Eq. 1 in the text; (c) a transmission spectrograph using a holographic grating. Both types of spectrographs are used in this study.

We begin with the relationship between the wavelength of a point-source at the spectrometer slit, to the position of the image of this point on the array detector. The derivation of this expression has recently been published in Hennelly and Liu^29^ and so we will present only a summary here. The derivation is general for both transmission and reflection gratings, and the calibration algorithm that builds on this and which appears in the next section can, therefore, be applied to spectrometers that employ both types of gratings. The relationship between the wavelength, λ , and the position, x , on the detector plane is given by:

(1) $$x=\frac{\;f}{T}\tan\;\left\{\theta_{d}+\sin^{-1}\;\left[\frac{n\lambda }{d^{\prime }}-k\sin\;\left(-\alpha -\theta_{d}\right)\right]-\alpha \right\}+\frac{C}{T}$$

where n is the diffraction order, f is the focal length of the spectrometer, T is the pixel pitch of the detector, $\theta_{d}$ represents the angle of the grating, k is an integer with value −1 for a transmission grating and +1 for a reflection grating, α is half the deviation angle of the spectrometer, and C represents misalignment of the center of the detector array with respect to the optical axis. The curvature of the slit image in the detector plane is often caused by the displacement of the irradiance spot vertically along the slit resulting in an oblique angle of the light, γ , incident on the grating,^30–33^ and $d^{\prime }=d\cos\;\gamma$ , where d is the grating period. We refer the reader to Figure 1 in Hennelly and Liu^29^ for an illustration of the rotating grating, which includes each of these parameters.

### Relationship Between Wavenumber Shift and Pixel Position for a Generalized Spectrometer with a Rotating Grating

The wavenumber conversion formula is defined in Eq. 2 below:

(2) $$v=\left(\frac{1}{\lambda_{L}}-\frac{1}{\lambda }\right)10^{-2}$$

where the wavenumber shift, v , is defined in units of cm^−1^ and wavelength is defined in terms of meters and $\lambda_{L}$ denotes the laser wavelength. To define the relationship between wavenumber shift and pixel position this wavenumber conversion formula is applied to Eq. 1. The forward and inverse relations are defined in Eqs. 3 and 4 as follows:

(3) $$\begin{array}{r} \begin{array}{cc} x= & \frac{\;f}{T}\tan\;\{\theta_{d}+\sin^{-1}\;[\frac{\nu \lambda_{L}}{d^{\prime }(1-10^{2}\nu \lambda_{L})}-k\sin\;\left(-\alpha -\theta_{d}\right)]\\ & -\alpha \}+\frac{C}{T} \end{array} \end{array}$$

(4) $$\begin{array}{cc} v= & \frac{-10^{-2}n}{d^{\prime }\{\sin\;\left[\tan^{-1}\;\left(\left(xT-C\right)/\;f\right)+\alpha -\theta_{d}\right]+k\sin\;(-\alpha -\theta_{d})\}}\\ & +\frac{10^{-2}}{\lambda_{L}}\; \end{array}$$

In these two equations, the position on the detector, x , is defined in units of pixels. Eq. 4 is used as the basis of the wavenumber calibration algorithm that is outlined in a previous section. Before deriving this algorithm, we first explore the relationship between x and v for two different Raman spectrometers, which are illustrated in Figure 1, and which are later used for experimental validation of the proposed algorithm. The purpose here is to examine the nonlinearity of this relationship for different systems, in an effort to elucidate the inconsistent results presented in the literature to date on the optimal polynomial order to best relate x and v for different optical systems. It is important to point out that although this analysis will indicate that continually increasing the polynomial order will reduce the error of the fit of the x–v relationship, we have found that applying a polynomial order >3 to the reference lines in a wavenumber reference spectrum will not improve the wavenumber calibration error; conversely it will increase it due to the error in estimating the true peak sub-pixel position and resulting over-fitting to this erroneous data.

Full details of these two spectrometers are provided in Hennelly and Liu.^29^ For the purpose of this paper, it suffices to point out some key details and to provide the set of parameters for each system in Table I. The Czerny–Turner spectrograph (Andor Shamrock 500; SR-500i-A; Andor) is illustrated in Figure 1b; it used parabolic mirrors with a focal length of 500 mm and contains a motorized rotating grating with interchangeable reflection gratings of period, 300, 600, and 1000 lines/mm. A transmission spectrometer (HOLOSPEC-F/1.8I-VIS; Andor) is also investigated in this study, illustrated in Figure 1c. This lens-based system used volume-phase holographic transmission grating with 2455 lines/mm. The same detector is used for both cases: a cooled charge-coupled device (CCD; Andor iDus; DU420A-BR-DD; Andor) with 256×1024 pixels with a pixel-pitch, T of 26 μ m Further details on both spectrographs are available in Hennelly and Liu.^29^ The set of parameters that describe the Raman spectrometers are provided in Table I. These parameters are required for the algorithm that is proposed in an earlier section.

**Table I.** The parameters for the two spectrometers illustrated in Figure 1, which are investigated in this study. The laser wavelength was measured using a neon lamp.

Parameter	Unit	Czerny–Turner	Transmission
Reflection/transmission ( k )	NA	+1	−1
Diffraction order ( n )	NA	−1 300	+1
Grating period (days)	lines/mm	600 1000	2455
Half the deviation angle ( α )	degrees	10.94 4.8	45
Grating angle ( $\theta_{d}$ )	Degree	10.2 17.2	0
Focal length ( f )	mm	500 ∼ 4190	85
Bandwidth	cm^−1^	∼ 2696 ∼ 1650 ∼ 4.09	∼ 2430
Wavenumber Interval	cm^−1^	∼ 2.63 ∼ 1.61 9.73	∼ 2.37
Average resolution	cm^−1^	5.05 3.19	5.51
Camera pixel pitch ( T )	μ m	26	26
Camera width ( N )	pixels	1024	1024
Camera centre position ( C )	pixels	0	0
Laser wavelength ( $\lambda_{L}$ )	nm	532.11	532.11

NA: numerical aperture.

**Figure 2.**
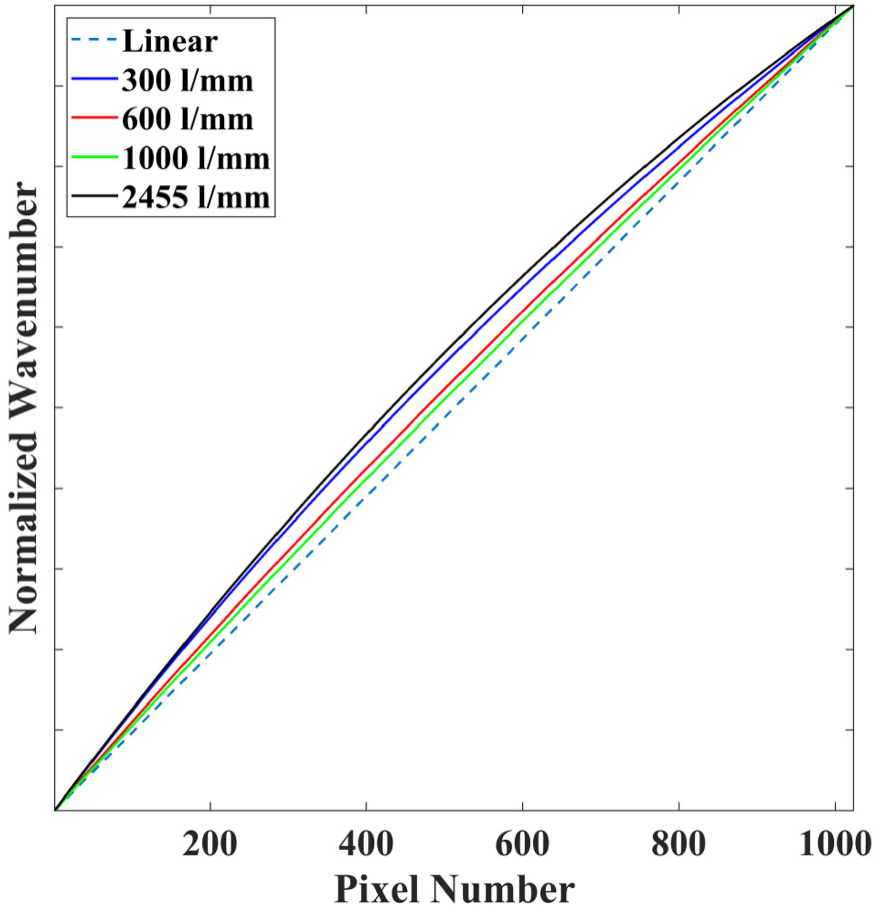
Investigation of the nonlinearity of the (x,v) relationship for the different spectrometers. Wavenumber shift values are normalized over the span of the detector for comparison.

In order to elucidate the nonlinear relationship between wavenumber shift and pixel position for these Raman spectrometers, Eq. 4 is plotted for integer values of x in the range −N/2→N/2−1 , as shown in Figure 2. The wavenumber shift values have been normalized for comparison in the figure. It is clear that the Raman spectrometer containing the transmission spectrograph with the short focal length and high dispersion grating (2455 lines/mm) exhibits the most nonlinear relationship over the span of the detector. Interestingly, for the spectrometer with the Czerny-Turner spectrograph, the most linear profile belongs to the case of the 1000 lines/mm grating, and the profile becomes more linear as the grating period reduces.

## Algorithm

In this section, an algorithm is developed that relates the detector pixels, x , to the corresponding spectral wavenumber shift values, v , which replaces the step of polynomial fitting in traditional direct wavenumber calibration algorithms. This algorithm is based on Eq. 4, which mathematically relates x and v using the physical model of the system. Like traditional polynomial fitting, this algorithm requires a set of matching sub-pixel positions, $X_{0}$ , and reference wavenumber shift values, $V_{0}$ , on which to apply the fitting algorithm. Following the recording and processing of a reference wavenumber spectrum, such a set of matching pairs will be available. Explicitly, these are defined as follows:

(5) $$\begin{array}{c} X_{0}=[x_{1},x_{2},\ldots ,x_{M}]\\ V_{0}=[v_{1},v_{2},\ldots ,v_{M}] \end{array}$$

where $x_{i=1\;:\;M}$ are the sub-pixel positions of the spectral lines, which have known reference wavenumber shift values $v_{i=1\;:\;M}$ , where there are M useful spectral lines in the reference spectrum.

Equation 4 contains several system parameters that define the system and knowledge of their precise values enables accurate fitting of Eq. 4 to the available data points $X_{0},V_{0}$ . The algorithm searches over a range of values of these system parameters to provide optimal fitting. The set of relevant system parameters, S is given by Eq. 6.

(6) $$S=[f,T,C,d^{\prime },\alpha ,\theta_{d},\lambda_{L}]$$

Each of these parameters is approximately known based on manufacturer specifications or approximate measurements. However, the precise values cannot easily be determined at the outset. Even the effective detector pixel size could be slightly reduced by a small out-of-plane tilt of the detector. To facilitate the discussion that follows, Eqs. 3 and 4 are rewritten using operator notation as follows:

(7) $$\begin{array}{c} x=\mathrm{model}(S,v)\\ v={\mathrm{model}}^{-1}(S,x) \end{array}$$

The goal is to find values of S that “best” match $X_{0}$ and $V_{0}$ . One naive approach is to perform a brute-force search over all seven parameters using their approximate values as a starting point; however, this approach is computationally intractable. Instead, it is sufficient to brute-force search only over a reduced set of four parameters $S^{\prime }=[d^{\prime },\alpha ,$ $\theta_{d},\lambda_{L}]$ , which significantly reduces the scope of the search. This is possible because the other three parameters, C , T , and f are linear in effect in Eq. 3, and can, therefore, be accounted for using linear regression with the brute-force search. The algorithm is defined in more detail as follows:

1. The first step provides initial estimates of the key parameters in S , which are defined as $S_{0}=[f_{0},T_{0},C_{0},d_{0}^{\prime },\alpha_{0},\theta_{d0},\lambda_{L0}]$ as follows: The values of ( $\;f_{0},T_{0},d_{0}^{\prime },\lambda_{L0}$ ) can be taken from the manufacturers specifications for the spectrograph detector, and laser. Note that approximate values can be used here: for example, it is not necessary to know the laser wavelength precisely, and defining it to the nearest nanometer is sufficient. For our experiments, the value for $\alpha_{0}$ was approximately measured by opening the spectrograph and visually inspecting the distance between the mirror centers and grating. We have found that defining α to within $\pm 2^{\circ }$ is sufficient for the algorithm to converge to the correct value. Both $C_{0}$ and $\theta_{0}$ can be estimated using a simplified brute-force search over only these two variables using a single peak pair $\left(x_{i},v_{i}\right)$ from the wavenumber reference spectrum, where the other parameters are defined as above.
2. A brute-force search is performed over $\left[\alpha ,d^{\prime },\theta_{d},\lambda_{L}\right]$ in a small range, centered at $\left[\alpha_{0},d_{0},\theta_{d0},\lambda_{L0}\right]$ and using values $f_{0},T_{0},C_{0}$ in order to provide an intermediate result:
(8) $$X_{j}=\mathrm{model}([C_{0},f_{0},T_{0},\alpha_{j},d_{j}^{\prime },\theta_{dj},\lambda_{Lj}],V_{0})$$
  For each unique set of values $\left[\alpha_{j},d_{j}^{\prime },\theta_{dj}\;\lambda_{Lj}\right]$ over the search range, the resultant values $X_{j}$ are linear-regressed with respect to positions at which the peaks were detected, $X_{0}$ , in order to account for errors in f , T , and C , which provides an updated set of estimated positions, $X_{j}^{\prime }$ . For simplicity, we describe this operation in terms of the Matlab functions, polyfit and polyval, which are used to implement it as follows:
(9) $$\begin{array}{c} P_{j}=\mathrm{polyfit}(X_{j},X_{0},p)\\ X_{j}^{\prime }=\mathrm{polyval}(X_{j},P_{j}) \end{array}$$
  where the function polyfit returns the coefficients of polynomial degree p=1 that is the best fit (in a least-square sense) to describe the transformation between $X_{j}$ and $X_{0}$ . This is followed by the function polyval, which applies this transformation to $X_{j}$ using these coefficients in order to provide the updated values for $X_{j}^{\prime }$ .
  The specific set of parameters, $S_{\min}=[f_{0},T_{0},C_{0},$ $\alpha_{\min},d_{\min}^{\prime },\theta_{\mathrm{dmin}},\lambda_{\min}]$ , and linear regression coefficients defined by $P_{\min}$ , which produce the set $X_{\min}$ that most closely match the actual pixel values $X_{0}$ are taken to be the system parameters. This is determined by minimizing the error function defined in Eq. 10:
(10) $$err=\sum_{i=1}^{N}(x_{i}^{j}-x_{i}{)}^{2}$$
  where $X_{j}^{\prime }=[x_{1}^{j},x_{2}^{j},\ldots ,x_{M}^{j}]$ . We acknowledge that a tight-grid brute-force search over four parameters would not normally be applied since modern search algorithms are far more efficient such as steepest descents and simplex searching. However, for the purpose of this paper, a brute-force search was sufficient.
3. Now that the system parameters $S_{\min}$ and $P_{\min}$ are known, the integer pixel (center) positions can be related to the corresponding wavenumber shift values, V , thereby providing wavenumber calibration for the spectrograph. In this final step, the pixel positions are projected into the wavenumber domain by using the opposite process outline in Step 2. Taking the center of the CCD pixels to be given by $X_{\mathrm{CCD}}=[1,$ 2,…,1024] , the matching wavenumber shift values are given by:
(11) $$V_{\mathrm{CCD}}={\mathrm{model}}^{-1}[S_{\min},\mathrm{polyval}(X_{\mathrm{CCD}},P_{\min})]$$

The algorithm is general for any spectrometer; however, the constant integer values of n and k that are used in Eq. 4 should be chosen accordingly. In our experiments, the transmission spectrometer uses the n=1 diffraction order, while the Czerny–Turner system uses the n=−1 diffraction order. The value of k depends on the use of a reflection or transmission grating. Therefore, for the case of the transmission spectrometer k=−1 , while for the Czerny–Turner system k=1 as shown in Table I. The Matlab code for the algorithm is available from Liu.^34^

**Figure 3.**
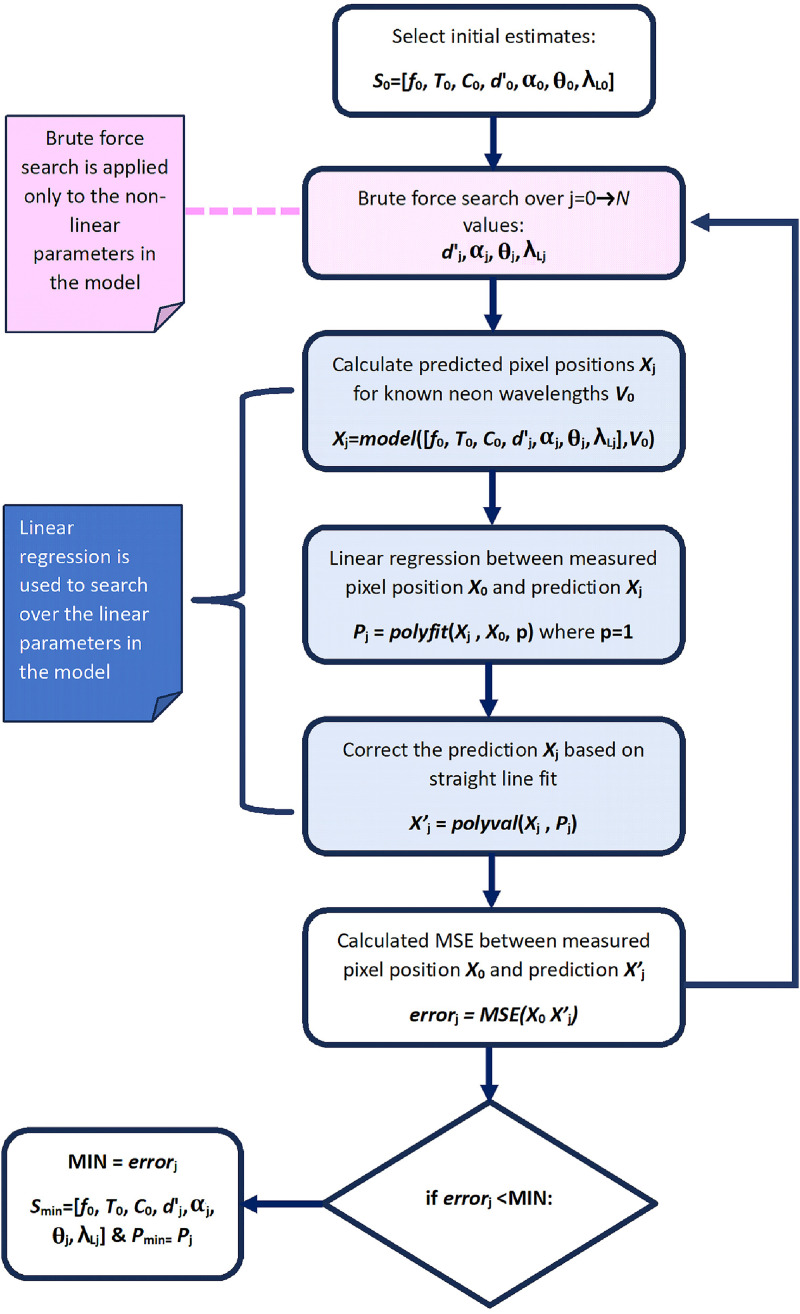
This flowchart illustrates the algorithm. See text for further details.

The flowchart in Figure 3 illustrates the key components of the algorithm. In summary, the first step is to select estimates of the seven parameters that describe the model. In the model, three of the parameters can be identified as linearly relating the wavelength and pixel positions, while the other four are nonlinear. The next step is to perform a brute-force search over only these nonlinear parameters in the form of four nested for loops using a range of values in the neighborhood of the initial estimates. In total, we will search over N possible sets of values of these four parameters, and each set of values is uniquely identified with an index j . Within these nested for loops, the predicted pixel positions $X_{j}$ are calculated using the model, and the linear parameters can then be effectively searched for using a linear regression over the prediction $X_{j}$ and the measured pixel positions $X_{0}$ . Based on this linear fit, the prediction can be refined to give $X_{j}^{\prime }$ . The set of parameters j that produces a refined prediction $X_{j}^{\prime }$ that most closely matches the measured positions $X_{0}$ is determined using a mean square error approach and this set of parameters is taken to define the model.

## Overall Calibration Procedure

**Figure 4.**
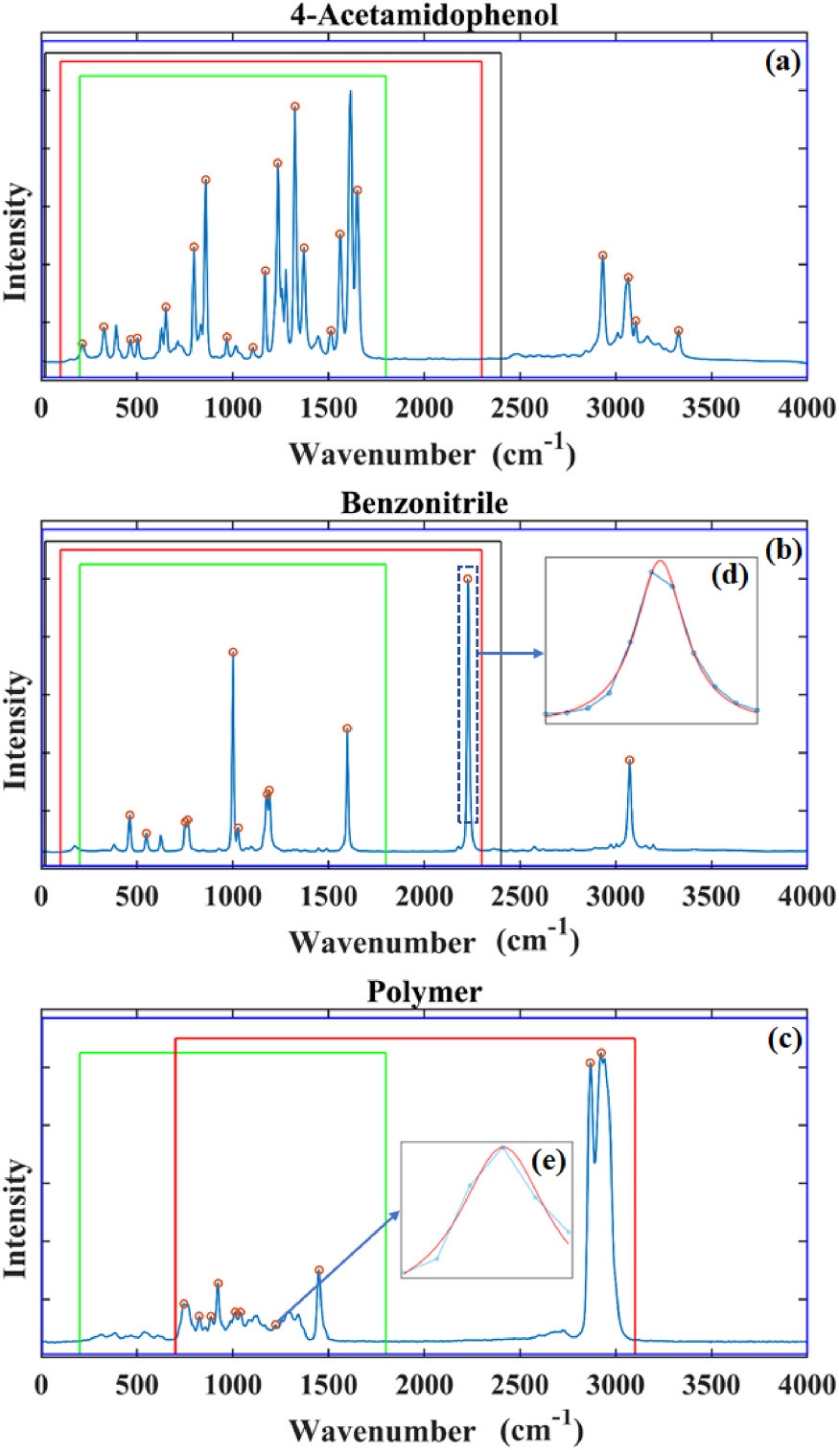
Sample spectra recorded from the three different reference materials. The spectrum of (a) 4-acetamidophenol, (b) benzonitrile, and (c) commercial-grade polymer. The recorded bands using the four spectrometers are highlighted in different color boxes: the black area corresponds to the 2455 lines/mm grating; green is 1000 lines/mm; red 600 lines/mm; blue 300 lines/mm. (d) A single peak from the benzonitrile is expanded. A Lorentzian function is fitted to the data points around the peak in order to detect the peak center with sub-pixel accuracy. (e) A further example is given of Lorenzian peak fitting, this time applied to a relatively broader and weaker peak.

The overall calibration protocol is similar to that outlined in Hennelly and Liu^29^ with some additional steps. Step 1 is to record a Raman spectrum from a reference material containing some number of sharp, symmetrical, and well-defined, known peak wavenumber shifts. Here, we use three reference materials: 4-acetamidophenol (Sigma), benzonitrile (Sigma), and a commercial polymer slide ( μ Slide I Luer, Ibidi GmbH) that is commonly used for life science purposes. This slide is designed with a flow channel for imaging adherent cells under flow conditions as well as in 3D cell culture. The base, which we target for RS, is composed of a transparent polymer with coverslip thickness and has favorable properties for imaging including a refractive index that matches that of glass; the specific chemical structure of the polymer material is proprietary, and could not be ascertained for this paper. This polymer was selected for study due to its ease of mounting, photothermal stability when used with laser illumination, and the high number of reproducible Raman spectral lines that can be used for wavenumber calibration.^35^ The slide chamber was also found to be useful for housing the 4-acetamidophenol powder and the benzonitrile. We note that other polymers could also potentially be used for wavenumber calibration including polytetrafluoroethylene (PTFE) and poly(methyl methacrylate), which although contain relatively few sharp peaks, could potentially be used with the proposed algorithm in this paper. Recorded spectra are shown in Figure 4, which also illustrates the different bands that were recorded using the four different spectrometers.

The wavenumber shift values of the lines that are highlighted in Figure 4 are provided in Table III. These reference values and uncertainties have been taken from ASTM-1840^23^ and Liu et al.^35^ For the case of the 300 lines/mm grating, all of the values shown in Table III were used for wavenumber calibration and a reduced set was used for the other gratings as detailed in the caption for Table III.

Step 2 is to identify the sub-pixel position of the lines in the recorded spectrum. Various methods are reviewed in the background section in the Supplemental Information on how this can be achieved; here, we fit a Lorentzian function to the intensities of the pixels in the region of the peak of the following form:^36^

(12) $$\frac{P_{1}}{(x-P_{2}{)}^{2}+P_{3}}+P_{4}$$

where $P_{1}$ , $P_{2}$ , $P_{3}$ , and $P_{4}$ are the fit parameters. The value of $P_{2}$ is taken to be the sub-pixel position of the peak. An example of this approach is given in Figure 4d in which we show a Lorentzian function that has been fit to one peak in the benzonitrile spectrum. In the Results section, all of the peak positions in each reference spectrum are estimated with sub-pixel accuracy using this approach. Step 3 is the application of the algorithm described earlier using these sub-pixel positions and the matching wavenumber shift values given in Table III.

## Experiment Design

### Recording of Reference Spectra

The performance of the proposed wavenumber calibration algorithm is examined across two Raman spectrometer designs and four different gratings periods as described earlier. For the case of the Czerny–Turner system, all three reference materials were investigated, while for the transmission spectrometer, the polymer was omitted. For each of the three gratings in the Czerny–Turner spectrometer, and for each of the three materials, 100 different reference spectra are recorded with changes in the grating rotation angle. For each of these cases, a rigorous evaluation of the performance of the calibration is possible by calculating the ensemble average of the error metrics defined below, across the set of 100 reference spectra.

While the polymer slide has the advantage of requiring no preparation whatsoever, the 4-acetamidophenol and benzonitrile are in powder and liquid form, respectively. These were both mounted in an Ibidi chamber slide (GmbH). The base of the chamber was drilled to create an open aperture, which was sealed using a Raman grade calcium fluoride coverslip (Crystran), which produces a negligible Raman spectrum except for a single peak at 321 cm^−1^.

To minimize the effect of shot noise, the accumulation time was maximized to provide a photon count just less than the saturation level of the CCD. Rather than use full vertical binning, which can produce errors in the presence of image distortion,^29^ area scan images were recorded by the detector; the row of pixels containing the spectrum was cropped.

### Error Metrics

Several different error metrics have been reported in the literature (see the Background section in the Supplemental Material) including, mean absolute error (MAE), the SD, and the root mean square error (RMSE), all of which are measured in this paper. These metrics are defined below using the same notation as used in the Algorithm section. Initially, we define the error for a single line at known wavenumber shift $v_{i}$ to be given by:

(13) $$\mathrm{error}(v_{i})=\mathrm{calibrated}(v_{i})-v_{i}$$

where the $v_{i}$ value is taken from Table III and the calibrated value, $\mathrm{calibrated}(v_{i})$ , is taken from the set $V_{\min}$ :

(14) $$V_{\min}={\mathrm{model}}^{-1}[S_{\min},\mathrm{polyval}(X_{\min},P_{\min})]$$

In some papers, the absolute error is reported for one peak or for a range of peaks. More traditionally, the mean error is reported for all of the peaks in the reference spectrum as follows:

(15) $$\mathrm{MAE}=\frac{1}{M}\sum_{i=1}^{M}|\mathrm{error}(v_{i})|$$

(16) $$\mathrm{RMSE}=\sqrt{\frac{1}{M}\sum_{i=1}^{M}|\mathrm{error}(v_{i}){|}^{2}}$$

(17) $$\mathrm{SD}=\sqrt{\frac{1}{M-1}\sum_{i=1}^{M}|\mathrm{error}(v_{i})-\bar{\mathrm{error}(v_{i})}{|}^{2}}$$

where $\bar{\mathrm{error}(v_{i})}$ denotes the mean error. In order to provide a more reliable estimate of the error, the above metrics can be calculated for a set of K different spectra where the grating is moved between captures. The ensemble average of each of the above metrics is calculated over these K reference spectra as follows:

(18) $$\bar{\mathrm{MAE}}=\frac{1}{K}\sum_{k=1}^{K}\mathrm{MAE}(k)$$

(19) $$\bar{\mathrm{RMSE}}=\frac{1}{K}\sum_{k=1}^{K}\mathrm{RMSE}(k)$$

(20) $$\bar{\mathrm{SD}}=\frac{1}{K}\sum_{k=1}^{K}\mathrm{SD}(k)$$

### Evaluation Methods

Here, we describe a number of evaluation methods, making use of the above metrics, which we recently proposed for the evaluation of wavelength calibration.^29^ The latter two are borrowed from the area of multivariate statistical analysis^37,38^ and are used here for the first time (to the best of our knowledge) in the evaluation of wavenumber calibration:

1. *All-peaks*: Here all of the calibrated peaks from the reference are used in the error analysis. This is the typical value reported in the literature to date. Taking a spectrum of the reference sample 4-acetamidophenol as an example, which contains 20 reference lines $v_{i}$ for i=1→20 , all 20 values of $v_{i}$ and the matching sub-pixel positions are used to perform the given calibration routine. Using the resultant calibrated wavenumber shifts, the error function for each $v_{i}$ is calculated according to Eq. 10, which enables the MAE to be calculated according to Eq. 15, and this procedure is repeated for each of the K=100 spectra in the data set; finally the mean MAE is calculated using 100 results as defined in Eq. 18. In total, a given calibration routine is applied 100 times to calculate the MAE for all peaks.
2. *Leave-one-out cross-validation (LOOCV)*: In order to remove any bias from the reference spectrum, we propose the use of “leave-one-out” (LOO) cross-validation, whereby one peak is removed from the reference spectrum used in the calibration process. The error metric is then applied only to this peak alone following calibration. This process is repeated for each peak in the spectrum and the average value for all cases is calculated. This method must provide a more accurate estimate of wavenumber shift accuracy *inside* the bounds of the spectral lines provided by the reference spectrum. Again taking a spectrum of the reference sample 4-acetamidophenol as an example, which contains 20 reference lines, the first line $v_{1}$ is removed from the spectrum and the given calibration routine is applied to the remaining 19 lines $v_{i}$ for i=2→20 . The resulting calibrated wavenumber axis is applied to the spectrum and the error function defined in Eq. 10 is applied only to $v_{1}$ to obtain $\mathrm{error}(v_{1})$ . Then $v_{2}$ is removed from the data set used for calibration followed by calculation of only $\mathrm{error}(v_{2})$ . This process is repeated in total 20 times to calculate $\mathrm{error}(v_{i})$ for all i=1→20 , and then the MAE function can be calculated as defined by Eq. 15. Therefore, LOO analysis applied to a single 4-acetamidophenol spectrum requires the application of the given calibration routine 20 times using a different set of 19 reference lines in each instance. This LOO analysis is then applied to all K=100 spectra in the data set and the mean MAE is obtained as defined in Eq. 18. In total, LOO analysis of a particular calibration routine requires 2000 applications.
3. *Leave-half-out (LHO)*: The purpose of this metric is to examine the impact of having a wide silent band on the left or right of the reference spectrum that is devoid of reference lines. We propose an evaluation based on calibrating using the left-most half of the reference peaks and applying the error metric to the right-most peaks of the calibrated spectrum. This is repeated using the right-most peaks for calibration and the left-most for error calculation. The average of the two values is taken. This provides a more accurate evaluation of the accuracy of the wavenumber calibration “outside” the bounds of the reference spectrum lines. Again taking a spectrum of the reference sample 4-acetamidophenol as an example, which contains 20 reference lines, the 10 left-most lines in the spectrum are removed and only the right-most 10 lines, $v_{i}$ for i=11→20 , are used in the given calibration routine. The resulting calibrated axis is applied to the full spectrum and the error function $\mathrm{error}(v_{i})$ is calculated only for the left 10 lines i=1→10 . This overall process is then repeated this time using the left 10 lines for calibration and the right 10 lines to calculate the error function. Thus, two applications of the given calibration routine, using 10 lines in each instance, will provide the 20 values of the error function, $\mathrm{error}(v_{i})$ for $v_{i}$ for i=1→20 , which can then be used to calculate the MAE function in Eq. 15. This LHO analysis is then applied to all K=100 spectra in the data set and the mean MAE is obtained as defined in Eq. 18. In total, LHO analysis of a particular calibration routine requires 200 applications.

We note that the Matlab code for the evaluation methods described above is available from Liu^34^ as is sample data used in the experiments described below.

### Comparison with Traditional Methods of Wavenumber Calibration

In the results shown here, the proposed algorithm is compared with equivalent results from first-order up to a fourth-order polynomial. This analysis relates to the discussion at the end of Section on the nonlinear relationship between x and v .

## Results and Discussion

**Figure 5.**
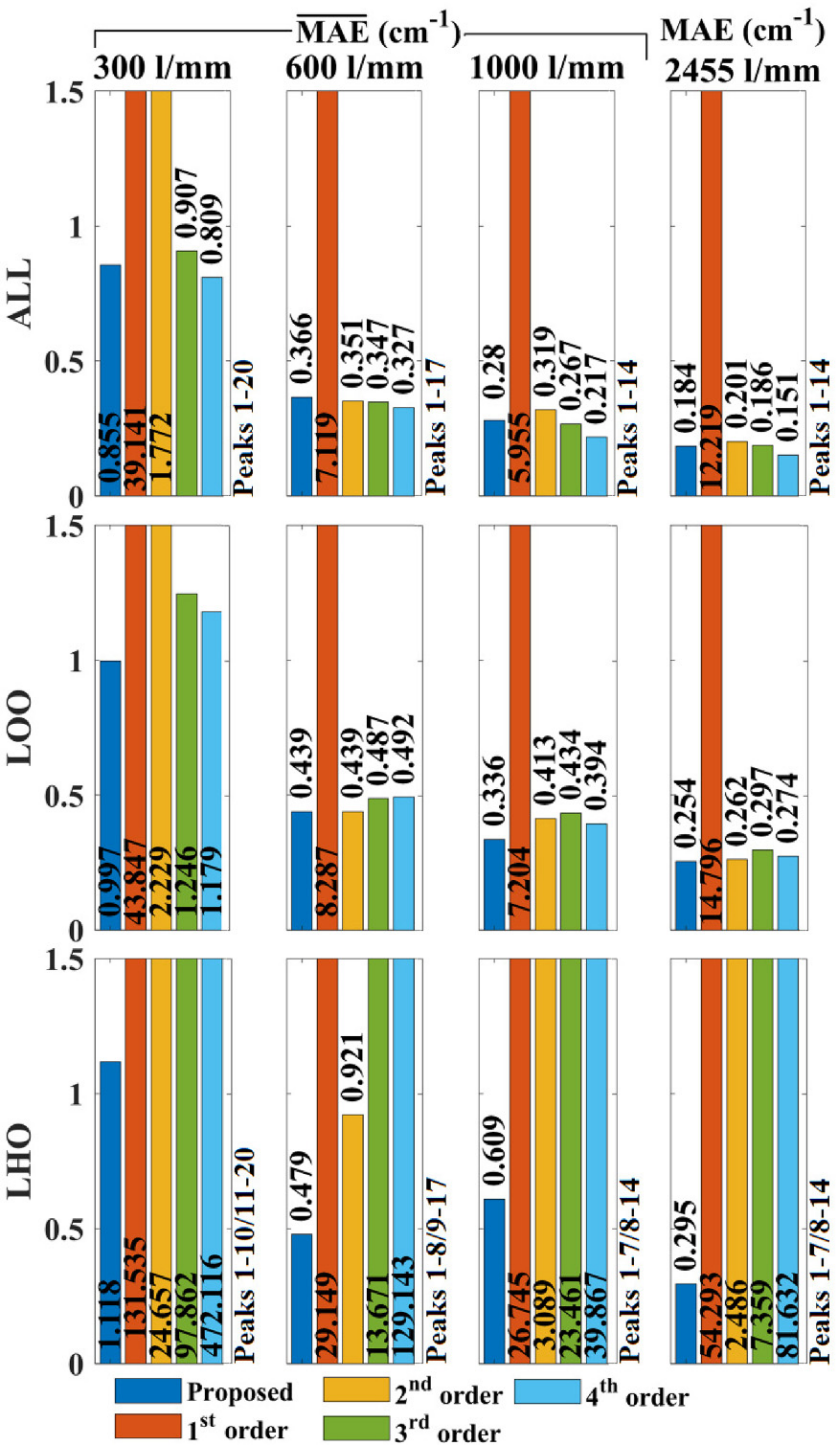
Evaluation of direct wavenumber calibration accuracy using MAE applied to 4-acetamidophenol spectra. For the case of the Czerny–Turner reflection spectrometer, three different gratings are investigated: 300, 600, and 1000 lines/mm, and for these three cases the $\bar{\mathrm{MAE}}$ error metric is applied over 100 spectra with grating movement between capture. The transmission spectrometer with grating 2455 lines/mm is evaluated using a single MAE error metric applied to a single spectrum. The results of the algorithm proposed in this paper are given in blue and the results for first-, second-, third, and fourth-order polynomial fitting are given in orange, yellow, green, and blue, respectively. The results of ALL LOOCV, and LHO are shown on different rows. For ease of comparison, the same axis range is used for all three evaluations. In several cases, the bars have been capped at 1.5 cm^−1^ to improve visualization. The correct values are overlaid on the bars in all cases.

In this section, the results are presented for wavenumber calibration using the proposed algorithm and compared with the corresponding set of results from first-, through to fourth-order (where possible) polynomial fitting. Polynomial orders of 5 and 6 were also tested but are not shown here due to the high error values. As outlined in the previous section, these results are broken down into three sets of evaluations, corresponding to “All-Peaks” (ALL), “LOO” cross-validation, and “LHO” cross-validation. Furthermore, to facilitate comparison with other papers, which use various metrics, these evaluations are performed using three different metrics: $\bar{\mathrm{MAE}}$ , $\bar{\mathrm{RMSE}}$ , $\bar{\mathrm{SD}}$ as defined in Eqs. 18, 19, and 20. For the case of the transmission spectrometer, the grating angle could not be adjusted and so only a single spectrum was available. In this case, the error metrics used in the evaluation are: MAE , RMSE , SD as defined in Eqs. 15, 16, and 17. The results for the MAE metrics using the 4-acetamidophenol material are shown in Figure 5. For the case of ALL evaluation, first-order fitting is the worst performer by a wide margin for all four systems, which is due to the highly nonlinear x – v relationship; the most inaccurate system is the 300 lines/mm grating, followed by the transmission 2455 lines/mm grating, which is predicted in Table II. We note that the measured error values differ from those in Table II due to the lower number of wavenumber shift values used to calculate the error when compared with the theoretical analysis in the earlier section describing the relationship between wavenumber shift and pixel position. For each of the four gratings, all of the other polynomial orders, as well as the proposed algorithm, show similar performance: the accuracy for the 300 lines/mm grating are 0.749–1.772 cm^−1^; for the 600 lines/mm grating the accuracy is 0.315–0.366 cm^−1^; for the 1000 lines/mm grating the accuracy is 0.177–0.319 cm^−1^; and for the 1000 lines/mm grating the accuracy is 0.140–0.201 cm^−1^. Interestingly, the proposed algorithm provides an accuracy equivalent to that of second- and third-order fitting, and the accuracy is improved slightly as the polynomial order is increased. The accuracy does not improve by an order of magnitude, with each increase in polynomial order as predicted by Table II; the limiting factor here is the positional accuracy afforded by sub-pixel interpolation, as well as the resolution of the given system.

**Table II.** The mean absolute error in units of cm^−1^ following the fitting of the profiles shown in Figure 2 with polynomials of orders 1–7. In this calculation, the wavenumber shift values have not been normalized.

Polynomial	300	600	1000	2455
Order	l/mm	l/mm	l/mm	l/mm
1	93.1	27.6	10.4	40.9
2	6.8	1.1	$2.2\times 10^{-1}$	$9.8\times 10^{-1}$
3	$4.9\times 10^{-1}$	$3.9\times 10^{-2}$	$4.3\times 10^{-3}$	$3.2\times 10^{-2}$
4	$3.6\times 10^{-2}$	$1.5\times 10^{-3}$	$8.7\times 10^{-5}$	$2.0\times 10^{-3}$
5	$2.6\times 10^{-3}$	$5.5\times 10^{-5}$	$1.8\times 10^{-6}$	$5.6\times 10^{-5}$
6	$1.9\times 10^{-4}$	$2.1\times 10^{-6}$	$3.6\times 10^{-8}$	$5.6\times 10^{-6}$
7	$1.4\times 10^{-5}$	$7.7\times 10^{-8}$	$7.2\times 10^{-10}$	$1.1\times 10^{-7}$

**Table III.** Reference spectral lines and uncertainties used in this paper.

Wavenumber shift (cm^−1^) ± SD		
4-acetamidophenol		
213.3±1.77	329.2±0.52	465.1±0.30
504±0.60	651.6±0.50	797.2±0.48
857.9±0.50	968.7±0.60	1105.5±0.27
1168.5±0.65	1236.8±0.46	1323.9±0.46
1371.5±0.11	1515.1±0.70	1561.5±0.52
1648.4±0.50	2931.1±0.63	3064.6±0.31
3102.4±0.95	3326.6±2.18	
Benzonitrile		
460.9±0.73	548.5±0.82	751.3±0.74
767.1±0.59	1000.7±0.98	1026.6±0.81
1177.9±0.82	1192.6±0.56	1598.9±0.70
2229.4±0.39	3072.3±0.41	
Polymer		
743.5±0.56	828.0±0.90	886.6±0.54
923.1±0.15	1005.7±0.17	1041.7±0.68
1224.6±0.60	1449.3±0.30	2869.0
2914.0		

Values for 4-acetamidophenol and benzonitrile are taken from ASTM-1840,^23^ and values for the polymer are taken from Liu et al.^35^ Different numbers of reference lines were used for the different spectrometers depending on their bandwidth as illustrated in Figure 4. For 4-acetamidophenol: 300 lines/mm (20 peaks), 600 lines/mm (17 peaks), 1000 lines/mm (14 peaks), and 2455 lines/mm (14 peaks); benzonitrile: 300 lines/mm (11 peaks), 600 lines/mm (10 peaks), 1000 lines/mm (nine peaks), 2455 lines/mm (10 peaks), polymer: 300 lines/mm (eight peaks), 600 lines/mm (10 peaks), and 1000 lines/mm (eight peaks). The uncertainty for the lines of the polymer is based on the four seconds recording in Liu et al.^35^ The uncertainty for the latter two lines is not available.

It is notable that for the case of ALL, the MAE reduced as the polynomial order increased. This was also found to be the case for orders 5 and 6, not shown here. It is likely that the better accuracy for order >3 results from over-fitting of the available data points. This is suggested by the second evaluation, LOO, which eliminates the possibility of over-fitting; In this case, it is clear that increasing the polynomial order will in general result in increased error “within” the wavenumber band that is bounded by the reference lines. This trend continues for orders 5 and 6, not shown here. In all cases, the proposed algorithm provided the highest accuracy for LOO evaluation, albeit the error is only slightly lower than for the best polynomial fitting case, which is either the second- or third-order for each case.

Leave-half-out (LHO) evaluation reveals the strength of the proposed algorithm over traditional methods. This evaluation indicates that in all cases, the proposed algorithm is by far the most accurate in wavenumber bands that are “outside” of the spectral lines in the reference lamp; indeed the accuracy in these bands is only slightly less (0.04–0.273 cm^−1^) than the accuracy “inside” the bounds according to LOO evaluation. For the case of the 300 lines/mm reflection grating, the proposed algorithm provides the best LHO accuracy, with an error of 1.118 cm^−1^ and second-order fitting is next best with an error of 24.657 cm^−1^; second- and third-order fitting errors are 22.1 times and 87.5 times worse than the proposed algorithm, respectively. For the 600 lines/mm reflection grating, the proposed algorithm once again provides the best LHO accuracy with an error of 0.479 cm^−1^; second-, and third-order fitting provide errors are 1.9 times, and 28.5 times greater. For the third reflection grating of the period 1000 lines/mm, the proposed algorithm once again returns the best LHO accuracy with an error of 0.609 cm^−1^; second-, and third-order fitting provide errors that are 5.1 times and 38.5 times greater. For the transmission grating of the period 2455 lines/mm, the proposed algorithm once again returns by far the best LHO accuracy with an error of 0.295 cm^−1^; second-, and third-order fitting provide errors that are 8.4 times and 24.9 times greater.

**Figure 6.**
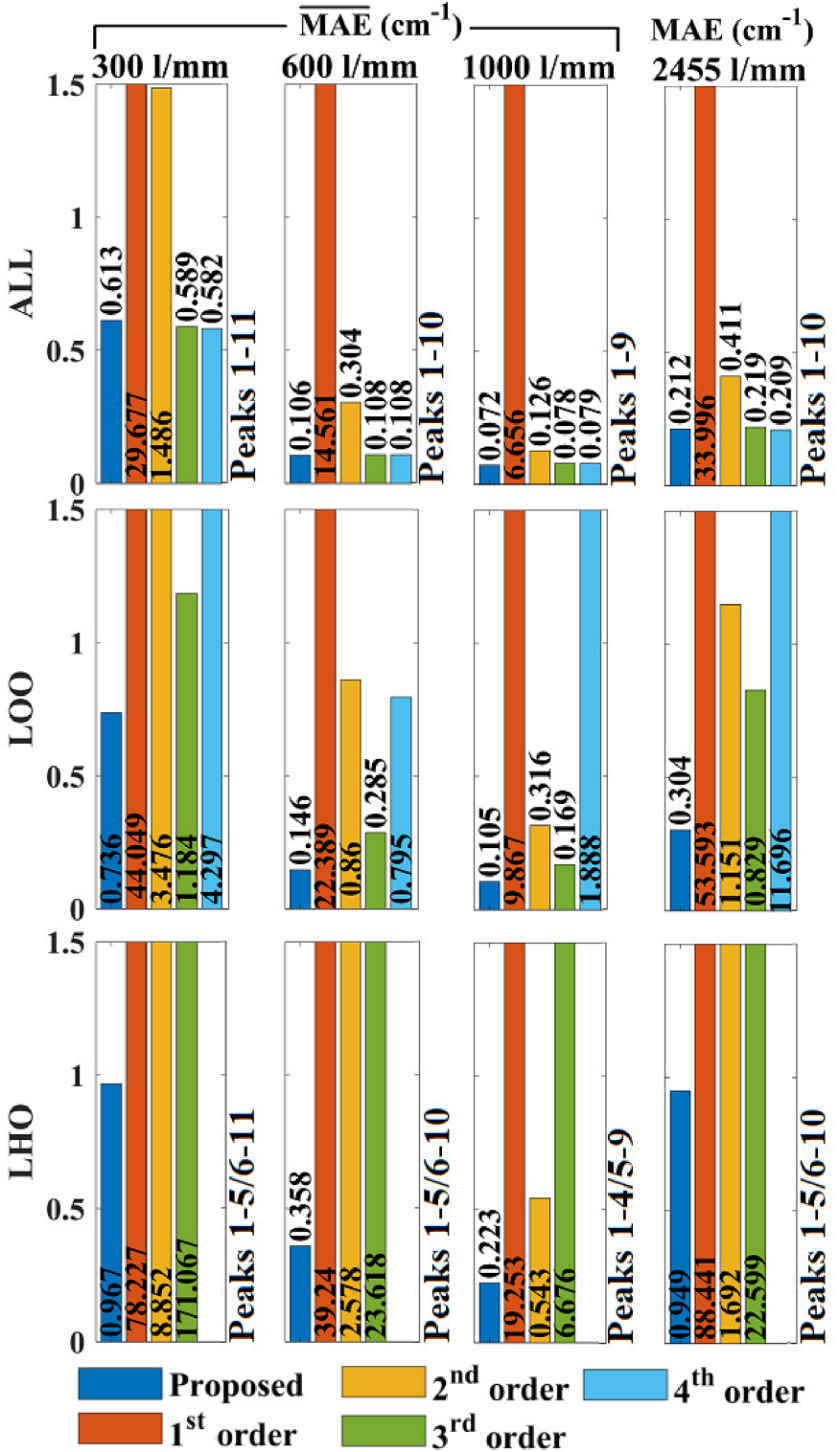
Evaluation of direct wavenumber calibration accuracy using MAE applied to benzonitrile spectra. See the caption of Figure 5 for further details. Polynomial order >3 could not be applied for LHO evaluation due to the lower peak number. The results of the algorithm proposed in this paper are given in blue and the results for first-, second-, third, and fourth-order polynomial fitting are given in orange, yellow, green, and blue, respectively.

Similar trends are reported for the benzonitrile reference spectrum as shown in Figure 6. In this case, it was not possible to perform polynomial fitting with order >3 for LHO evaluation owing to the availability of a smaller number of lines in the reference spectrum. Focusing only on LOO and LHO evaluation, which provides the best estimate of calibration accuracy inside and outside of the reference lines, it is notable that the proposed algorithm has the best accuracy in all cases, with the most pronounced improvement over polynomial fitting observed for LHO evaluation. For LOO evaluation, third-order fitting is the second most accurate in all cases; in summary, the proposed algorithm provides an accuracy of 0.105–0.736 cm^−1^ for the four spectrometers within the bounds of the reference lines, while third-order fitting produces errors that are 1.6–2.7 times greater. For LHO evaluation, second-order fitting is the second most accurate in all cases; in summary, the proposed algorithm provides an accuracy of 0.223–0.967 cm^−1^ for the four spectrometers outside the bounds of the reference lines, while second-order fitting produces errors that are 1.8–9.2 times greater, and third-order fitting produces errors that are 23.8–178.8 times greater.

**Figure 7.**
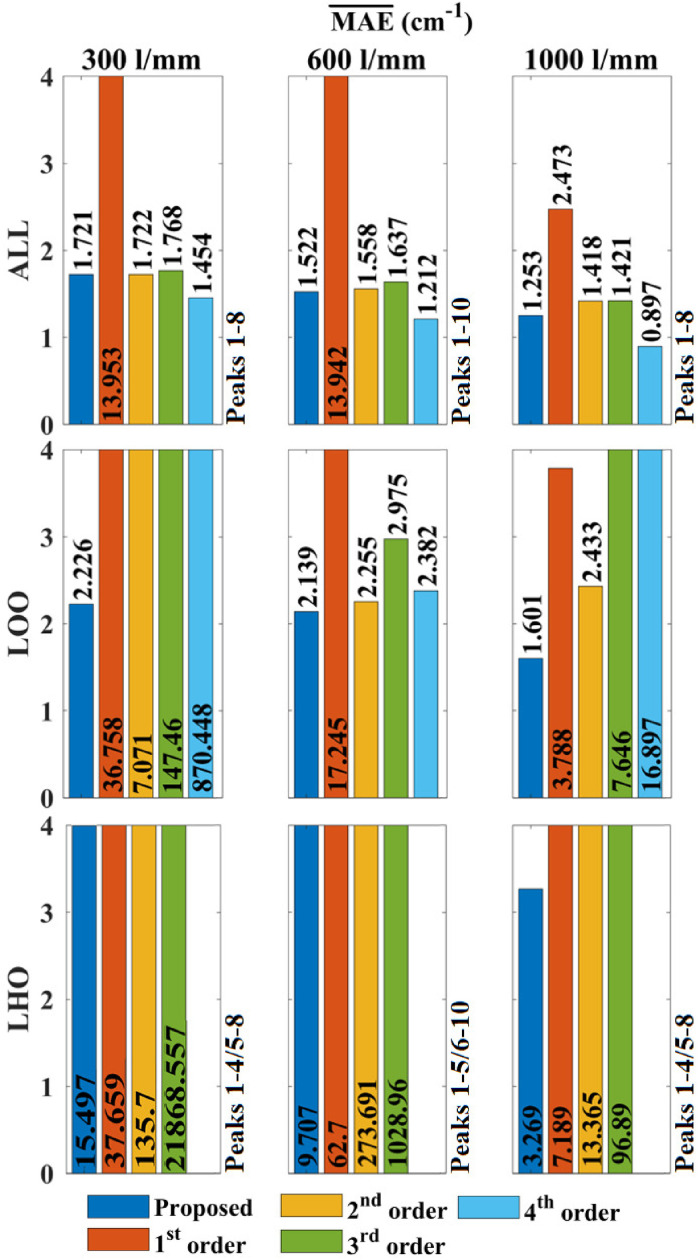
Evaluation of direct wavenumber calibration accuracy using MAE applied to commercial polymer spectra. In this case, the transmission spectrometer was not tested. Polynomial order >3 could not be applied for LHO evaluation due to the lower peak number. The results of the algorithm proposed in this paper are given in blue and the results for first-, second-, third, and fourth-order polynomial fitting are given in orange, yellow, green, and blue, respectively.

Although the polymer material has the advantage of photo-stability and easy mounting, it produces the least accurate wavenumber calibration results across the three materials tested. Nevertheless, it provides similar trends as for the other two cases as shown in Figure 7. As for benzonitrile, it was not possible to perform polynomial fitting with order >3 for LHO evaluation. Focusing again only on LOO and LHO evaluation, it is clear that the proposed algorithm has the best accuracy compared with the polynomial fitting of various orders, albeit the improvement over second-order fitting is minute for the 600 lines/mm grating. As before, the most significant improvement over polynomial fitting is observed for LHO evaluation. For LOO evaluation, second-order fitting is the second most accurate in all cases; in summary, the proposed algorithm provides an accuracy of 1.601–2.226 cm^−1^ for the three spectrometers within the bounds of the reference lines, while second-order fitting produces errors that are 1.1–3.2 times greater and third-order fitting produces errors that are 1.4–66.2 time greater. For LHO evaluation, first-order fitting is the second most accurate in all cases; in summary, the proposed algorithm provides an accuracy of 3.269–15.497 cm^−1^ for the four spectrometers within the bounds of the reference lines, while first-order fitting produces errors that are 2.2–6.9.0 times greater.

It is interesting to note that for the various reference materials, the accuracy of the ALL and LOO peaks analysis is of similar order to that predicted in Table II up to polynomial order of 3. It can be concluded that the limited precision in identifying the peak position prevents higher than third-order fitting.

In the Supplemental Information, we provide the same evaluations as above for the three materials, where the underlying metric of MAE is replaced with the SD, and the RMSE, which are sometimes preferred in the literature.

An additional result of interest relates to the laser wavelength. All of the results presented above relate to the initial input parameters to the algorithm taken from Table I except for the laser wavelength. The value input was 532 nm and not the measured value of 532.11 nm. The reason for this is that we wished to demonstrate that the algorithm could perform well using an approximate value of wavelength and could, therefore, be described as being more similar to direct wavenumber calibration methods as opposed to indirect methods that require precise measurement of the laser wavelength for wavenumber conversion. The value of wavelength that was calculated by the brute force component of the algorithm was accurate within 0.01 nm in all cases.

## Conclusion

In this paper, we have made several important contributions in the area of wavenumber calibration for RS. Firstly, in the Supplemental Information the background section provides a detailed review of direct wavenumber calibration in the literature and compares with the alternative approach of wavelength calibration followed by wavenumber conversion. In this review, we have compared the various contributions over several important features including the reference materials used, the number of peaks in the reference spectrum, the resolution of the systems, methods for sub-pixel interpolation, and calibration accuracy. We believe this is the first such review of its kind in the literature to date.

An important consideration is the derivation of the relationship between the detector pixel position x and wavenumber shift v for a low distortion Raman spectrometer, in terms of the system parameters including, laser wavelength, grating period and angle, and spectrograph focal length is provided. This relationship was explored for a number of different experimental systems, and it was demonstrated that the degree of nonlinearity was highly variable across the different systems and depended primarily on focal length and grating period; in some cases, a second-order fit could estimate the relationship with high accuracy, while in others this would result in a high error and a third order or higher polynomial order is necessary accurate fitting. We believe that this result goes some way to explain the variable results that have been reported in the literature to date on the optimal polynomial order to be used in direct wavenumber calibration.

The most significant contribution of the paper is the algorithm that can replace the polynomial fitting step applied in traditional wavenumber calibration to relate x and v . This algorithm searches for the optimal set of system parameters as functions of the x−v expression derived in the preceding section that can best explain the positions and wavenumber shift values for a set of spectral lines in a known reference spectrum. The algorithm can search over seven system parameters in total including the laser wavelength and is demonstrated to outperform traditional polynomial fitting in terms of a number of metrics.

In order to fully demonstrate the superiority of the proposed algorithm over polynomial fitting, we propose a number of novel evaluation methods, namely LOO and LHO cross-validation. Although these are well-known techniques in chemometrics, their applicability to wavenumber calibration has not previously been reported. We argue that these matrices are more suitable than traditional approaches as they preclude the possibility of over-fitting in the calibration process; by only allowing the accuracy to be measured on peaks that were not included in the calibration process, these methods must be considered to be a more accurate evaluation of the true accuracy of the calibration procedure at wavenumber shift positions between the spectral lines in the reference wave and in bands outside of the outermost peaks in the reference spectrum. Using these evaluations, we conclude that for the instruments tested here, the proposed algorithm is more accurate than second- or third-order fitting within the band of the spectral lines in the reference by a factor of up to 2.2 times for 4-acetamidophenol and 5.9 times for benzonitrile. More significantly it is more accurate than second- or third-order fitting outside of the reference lines by factors of up to 269.6 times and 176.9 times these two materials.

It is important to contextualize the use of the LOO and LHO metrics in the comparison of the proposed algorithm and traditional polynomial fitting. Our method outperforms polynomial fitting for both cases; for LOO there is only a slight improvement; however, for LHO the superiority of the proposed algorithm is much more significant. The LHO evaluation is somewhat of an edge case and it was expected that the proposed method would outperform polynomial fitting for this metric. Although the LHO metric somewhat exaggerates the error one can typically expect from polynomial fitting, we feel that this is a useful metric to highlight the advantage that can be provided by the proposed method in extreme bands. It is important to note that many wavenumber calibration materials have outermost peaks that do not extend to the end of the band of interest. As an example, for a spectrograph configured to record the fingerprint region 400–1800 cm^−1^, most reference materials will not extend beyond 1600 cm^−1^. Cyclohexane, for example, which is commonly used for wavenumber calibration would only cover the band from 426 to 1444 cm^−1^. In terms of wavenumber calibration accuracy, polynomial fitting cannot be trusted in this band. For this reason, some authors have used a combination of chemical spectra to increase the number and breadth of the available peaks for calibration REF The method proposed in this paper largely overcomes this limitation by fitting to the expected x – v characteristic for the given system. While we accept that the LHO metric exaggerates the advantage of the proposed algorithm, a much more detailed analysis of the error as a function of the “distance” from the outermost calibration peak is provided in the Supplemental Information, in which it is shown that the wavenumber calibration error from polynomial fitting can grow significantly even after only a few hundred wavenumbers from the outermost peak in the reference spectrum, while the proposed algorithm retains low error.

Another interesting conclusion is that benzonitrile provides for more accurate calibration than 4-acetamidophenol for all four spectrometers tested: the accuracy afforded by 4-acetamidophenol is LOO: 0.254–0.997 cm^−1^ and LHO: 0.295–1.118 cm^−1^ for the four systems, while for benzonitrile this drops to LOO: 0.105–0.736 cm^−1^ and LHO: 0.223–0.967 cm^−1^. The latter has significantly fewer peaks; however, these peaks are in general sharper, which may suggest a greater importance for peak width compared with peak number for wavenumber calibration. It is important to emphasize that the proposed algorithm negates the need for a large number of peaks. The accuracy when using only nine peaks for the case of the 1000 lines/mm grating is ALL: 0.072 cm^−1^, LOO: 0.105 cm^−1^, and LHO: 0.223 cm^−1^. These values compare well with the most accurate calibration reported to date,^39^ which was limited only by the accuracy of the ASTM values for the reference lines of 0.1 cm^−1^. In that paper, the authors used 67 peaks from a reference spectrum from a composite of different materials, chosen to cover a wide range in wavenumber. The proposed algorithm may negate the need for such an approach, and we believe there is a strong case for it to be included in future iterations of ASTM-E1840, in particular for wavenumber bands outside the range of lines in the reference spectrum.

At the heart of the proposed method is a nonlinear optimization scheme and the results from such procedures can be highly dependent on the quality of the provided initial values, and may not converge to the most optimum value if initial guesses are not reasonable. Further work is required to evaluate the impact of large errors in the initial parameter values. In the experiments described in this paper, we did not encounter any major issues in selecting the initial values that worked well; most of the system parameters are approximately known from manufacturer specifications, with the exception of the two parameters that relate to grating angles. In our case, we were able to measure these approximately after removing the cover from the spectrograph.

One of the assumptions in this paper is that the same physical model applies to transmission and reflection spectrographs; however, there exists a significant difference between the two types. For ruled reflection gratings, diffraction occurs at the interface between the air (with a refractive index that can be ∼ 1 for all wavelengths) and the grating material. For the volume phase transmission grating, diffraction occurs at the diffraction grating formed inside the dichromate gelatin film (with a refractive index that has a much larger wavelength dispersion than that of air). Therefore, for the volume phase grating, the change in the medium refractive index with wavelength results in a change in the apparent grating period. This difference between the true physical model and the one used as the basis for the algorithm proposed in this paper will affect the wavenumber calibration accuracy of the algorithm for the case of spectrometers using volume phase transmission. This is investigated further in the Supplemental Information, where our algorithm was applied to simulated data that included this additional dispersion. It was found that the resultant error had an MAE of 0.33 cm^−1^ with the value increasing with distance from the center pixel up to 1 cm^−1^ at the edge. Overall, these values are consistent with the experimental error measured for the Holospec spectrograph. It is notable that even though we do not attempt to account for the dispersion of the film in the underlying model, the proposed algorithm has comparable performance to polynomial fitting using the LOO evaluation and significantly superior performance for the LHO metric. Further work is required in order to account for the effect of dispersion in the algorithm, and potentially improve these results further for the case of volume phase transmission gratings.

We summarize the advantages of the proposed algorithm as follows: (i) The main advantage of the proposed algorithm for direct wavenumber calibration using a Raman reference wavenumber standard is that it outperforms traditional polynomial fitting in terms of accuracy, as demonstrated by novel evaluation methods such as LOO and LHO cross-validation. Although it is demonstrated to be superior in all wavenumber regions, it is especially so in regions outside of the range of peaks in the reference spectrum; (ii) an advantage over the common wavelength calibration/wavenumber conversion protocols, which are often preferable due to the highly reproducible atomic emission spectra with extremely sharp line shapes, is that the method has only a single step and does not require the wavelength of the laser to be carefully or accurately measured; an approximate estimate is sufficient; (iii) an advantage over direct wavenumber calibration protocols that can currently be found on some commercial instruments is the generality of the approach such that it can be applied to various spectrograph, reflection and transmission alike, even if the specifications of the device are not known exactly; only approximate values for the spectrograph parameters are required for the algorithm to converge. Disadvantages of the algorithm include (i) that it is slower than traditional polynomial fitting; the time taken to calibrate the using 20 peaks (4-acetamidophenol, 300 lines/mm) is 160 ms using polynomial fitting and 35 s using the proposed algorithm when processed using an i7 intel processor; (ii) an additional disadvantage is the error that is inherent in the method for the case of volume phase transmission holographic gratings, for which case the dispersion caused by the material of the grating cannot easily be accounted for. Nevertheless, the results still outperformed those for traditional polynomial fitting.

## Supplemental Material

sj-zip-1-asp-10.1177_00037028241254847 - Supplemental material for Wavenumber Calibration Protocol for Raman Spectrometers Using Physical Modelling and a Fast Search Algorithm Supplemental material, sj-zip-1-asp-10.1177_00037028241254847 for Wavenumber Calibration Protocol for Raman Spectrometers Using Physical Modelling and a Fast Search Algorithm by Dongyue Liu and Bryan M. Hennelly in Applied Spectroscopy
