# Supplementary material for: Wavenumber Calibration Protocol for Raman Spectrometers Using Physical Modelling and a Fast Search Algorithm
Source: Appl Spectrosc. 2024 Jun 2;78(8):790–805. doi: 10.1177/00037028241254847 (PMC11340246; doi:10.1177/00037028241254847)
Supplement: sj-zip-1-asp-10.1177_00037028241254847 - Supplemental material for Wavenumber Calibration Protocol for Raman Spectrometers Using Physical Modelling and a Fast Search Algorithm [file sj-zip-1-asp-10.1177_00037028241254847.zip › ASP-23-0305.R1_Hennelly_Supplemental_Material/supplementary_information.pdf]

Background - Wavenumber Calibration

There are two approaches to wavenumber calibration in Raman spectroscopy: (i) Initial wavelength calibration using an atomic emission spectrum such as neon (for a comprehensive review of wavelength calibration we refer the reader to Ref. 1), followed by wavenumber conversion making use of the excitation laser wavelength in this calculation; (ii) Direct wavenumber calibration using a Raman reference standard such as acetaminophen or indene. Both approaches appear to be equally represented in the literature and both require the use of a reference standard with a spectrum containing several well defined narrow peaks, which are subject to polynomial fitting. In Tables 1 and 2, a non-exhaustive literature review is provided of both approaches. This table compares the different reference standards, polynomial orders, sub-pixel interpolation methods, and accuracy of both approaches. To save space, the footnote of the tables are given in the following section.

In the first publication to examine a wavenumber calibration protocol,[2] Hamaguchi et al. reported that wavelength calibration followed by wavenumber conversion provided higher accuracy. However, our review of the literature reveals that both approaches provide similar levels of absolute error (accuracy) with a limit of approximately 0-0.1cm^{-1} and a standard deviation (or precision) of 0-0.1cm^{-1}. Direct wavenumber calibration is a single step approach with the advantage of not requiring *a priori* knowledge of the laser excitation wavelength, which can be difficult to record using the Raman system if the spectrograph grating is configured to avoid this wavelength, or if long-pass or notch filters remove it. Rotating the grating to record the laser also presents problems in that wavelength calibration must be repeatedly applied following rotation. In such cases, a separate spectrometer is sometimes used to record the laser wavelength. Alternatively, the laser wavelength can be estimated by measuring the Raman wavenumber shift of Raman reference standards such as cyclohexane.[3,4] Error in the laser wavelength measurement is problematic; for example, in Ref. 5, the authors report an uncertainty of $\pm$0.005 nm in the laser wavelength introduces an error of 0.18 cm^{-1} in the corresponding relative wavenumber shifts, at 532 nm excitation. Given the similar accuracy of the two approaches and the additional difficulties in the two step wavenumber conversion approach, it can be taken that direct wavenumber calibration is preferable.

Typically the wavenumber conversion approach uses neon as the reference material. However, the reference standards that have been investigated for direct wavenumber calibration are far more varied[22] as seen in Table 2. In Ref. 12 the authors define the characteristics of a suitable wavenumber reference standard. In summary, the spectrum must contain a large number of sharp peaks (of known wavenumber shift) that extend over the full range of the recorded bandwidth; the accuracy of the calibration outside of the end peaks drops significantly. Since no single standard will provide this, it is common to see several reference materials combined in a calibration protocol.[13,14] The sampling considerations of the standards is also important and should resemble those of the samples to avoid any change in the recording conditions. Gaseous samples can be troublesome as these requires specialised containers, although these samples typically provide the sharpest peaks. Also important is the long term stability of the sample; photo-degradation of the sample or chemical change over time can result in errors. One final note on the selection of wavenumber reference standards is on the use of a single peak standard such as Silicon.[23] Such a standard is sometimes used to account for a constant offset in the wavenumber shift axis due to small daily changes in the system/environment. It has recently been shown that this approach results in significant error;[19] it is notable that the European Pharmacopoeia describes the requirement of a minimum of three wavenumber shifts covering the working range of the instrument.[24]

A common feature in wavenumber calibration using either of the approaches is the identification of peak position with sub-pixel accuracy, as highlighted in the 'sub-pixel interpolation' column in Table 1 and 2. Any error in identifying these peak positions will consequently result in a calibration error. A desirable characteristic of the wavelength/wavenumber standards in that the peaks are narrow; however, this presents a problem for accurately identifying the position of the centre of peak if the sampling interval of the detector is of similar width or larger than the peak width. An early approach involved the recording of many spectra with very small shifting of the spectrum between captures.[25] To overcome the problem for a single spectrum, many authors use a first step of apodization,[4,6,7] whereby the raw spectrum is subject to a discrete Fourier transform (DFT), followed by multiplication with a Gaussian function or similar and an inverse DFT. The result is a convolution of the raw spectrum with a symmetrical blurring function, which broadens the shape of the peaks such that the centre can be more easily identified. The DFT is implemented with computational-efficiency using the fast Fourier transform (FFT) algorithm. An additional step of interpolation is commonly applied, whereby the DFT of spectrum is zeropadded to increase the number of samples n-fold, which provides n times interpolation. In the table this is denoted as $n\times$FFT. Finally, in order to identify the position of the peak with an resolution smaller than the sampling interval, the peaks are fitted using a Gaussian, Lorentzian, or Voigt function;[6,8,9,14,18,20,21] and the centre of the fitted function is taken to be the peak position. Alternatively, polynomial fitting[4,5,7,11,13,15] can be applied to the few samples around the maximum peak sample of the peak; the zero valued derivative of the polynomial in this region reveals the local maximum, which is taken as the peak position.

Once the position on the detector of each peak in the reference spectrum have been identified with sub-pixel accuracy, these values are then subjected to polynomial fitting with respect to their known wavelength or wavenumber shift values. In this way the wavelength or wavenumber shift axis for the spectrum is determined. For the former, the wavelength axis is then converted to wavenumber shift values making use of the laser wavelength. Although the first paper to investigate wavenumber calibration employed a polynomial order of 5 (necessitated

Wavelength Calibration followed by Wavenumber Conversion						
**Ref**	**Peaks**	**Poly-nomial order**	**Reference material**	**Sub-pixel interpolation**	**Resolution (cm^{-1})**	**Calibration accuracy (cm^{-1})**
2	21	3-5	neon	-	-	SD:0.2-0.4<br>AE:0.2-0.5
3	39/16	5	argon, neon [†1]	8×FFT	5-7	$\overline{\text{MAE}}$:0.85<br>$\overline{\text{SD}}$:0.257[†2]
6	5	1	neon	Apodization + 4×FFT + Gaussian fit	10	MAE:1<br>$\overline{\text{SD}}$:0.2
7	50	1[†3]	thorium, neon	Apodization + -×FFT + 4th order polynomial fit	4	$\overline{\text{SD}}$:0.04-0.06
4	59/21[†1]	1	neon [†1]	Apodization + -×FFT + polynomial fit	-	RMSE:0.4<br>$\overline{\text{SD}}$:0.05
8	9	-[†4]	neon	Gaussian fit	-	-
9	28	3	neon [†5]	Gaussian fit	1.8	$\overline{\text{SD}}$:0.6
10	15	2	neon, mercury	8×FFT + log-normal function fit	0.87-1.4	$\overline{\text{SD}}$:0.1
11	18/11[†6]	1	neon [†6]	-×FFT + polynomial fit	-	SD:0.24/0.04[†6]

Table 1. Non-exhaustive literature review of wavenumber calibration for Raman spectrometers using first wavelength calibration followed by wavenumber conversion. Methods are compared in terms of: reference materials used, number of peaks, polynomial order, method for sub-pixel interpolation to identify the peak positions on the detector with high accuracy, resolution of the systems, and the reported accuracy/precision. For the latter, the various metrics given here as abbreviations are defined in the main paper in a section on error metrics. To save space, footnotes are provided in the following section.

Direct Wavenumber Conversion						
**Ref**	**Peaks**	**Poly-nomial order**	**Reference material**	**Sub-pixel interpolation**	**Resolution (cm^{-1})**	**Calibration accuracy (cm^{-1})**
2	17	-	indene	-	-	-
7	5	1	acetonitrile	8×FFT + 4th order polynomial fit	4	-
12	17	1-3	indene	8×FFT	-	MAE:(1:0.25, 2:0.2,3:0.16)[†7]
13	67	4	4-acetamidophenol, benzene, polystyrenel, acetonitrile	64×FFT + 2nd order polynomial fit	4	AE:0-0.1<br>SD:0.01-0.21[†8]
14	45	3	H_2, HD, D_2, O_2	Gaussian, Lorentzian, or Voigt fit	3.5	MAE:0.08-0.11[†9]
5	20	1	CO_2, CO, N_2O, H_2O, HC1	4th order polynomial fit	0.06	MAE:0.003-1<br>$\overline{\text{SD}}$:0.002
15	15	4	4-acetamidophenol	3rd order polynomial fit	2.5	SD: 0.0374-0.6542[†10]
16	8	2-4	indene	-	-	-
17	19	2-4	4-acetamidophenol	-	4.36-12.03	RMSE: 0.52-3.56[†11]
18	13	3-5	4-acetamidophenol	Gaussian or Lorentz fit	-	-
19	3	-	polystyrene, benzonitrile, cyclohexane[†12]	-	-	nIQR:0.74[†13]
20	10/5/5	3	4-acetamidophenol, cycohexane, polystyrene	Gaussian fit	1-20	MAE:0.45-1.93<br>SD:0.24-2.16[†14]
21	-	3	acetone, sodium perchlorate, potassium perchlorate, acetonitrile, military C-4.	Gaussian fit	2-5	AE:0-30

Table 2. Non-exhaustive literature review of direct wavenumber calibration for Raman spectrometers. Methods are compared in terms of: reference materials used, number of peaks, polynomial order, method for sub-pixel interpolation to identify the peak positions on the detector with high accuracy, resolution of the systems, and the reported accuracy/precision. Footnotes are provided in next section.

by the presence of high levels of distortion in the imaging system), a low order polynomial is generally preferred in the literature for both the wavenumber conversion and direct wavenumber calibration approaches, as seen in

Tables 1 and 2. In Ref. 16, the authors compared the use of 2nd, 3rd, and 4th order polynomial fits. They demonstrate that 4th order fits give significantly worse results in comparison to 2nd and 3rd order fits. Their data also indicates that better fits were obtained with a quadratic equation for three (413.1, 487.9, and 514.5 nm) of the five excitation wavelengths examined. Interestingly, it has been shown that a quadratic expression is sufficient to model the relationship between wavelength and a CCD detector based on the physics of dispersion from a diffraction grating and imaging in the presence of low distortion; the coefficients of the second order polynomial can be defined in terms of the system parameters.[10] Recently, we have shown that the non-linearity of this relationship varies considerably as a function of grating period and focal length.[1] It can be expected that the non-linearity of the wavenumber shift axis will be more pronounced given the non-linear relationship between wavelength and wavenumber shift, and therefore, a polynomial order > 2 may be referred. We examine this point in more detail the main paper. It is notable that recent large scale cross-instrument investigations have preferred 3rd order polynomials for direct wavenumber calibration.[17,20,21] Some authors have employed linear interpolation between adjacent reference peaks as an alternative to polynomial fitting with high levels of accuracy.[7,11]

A key point that is often overlooked is the accuracy of the points used in the polynomial fitting; while great attention is given to finding the position of the peaks on the detector with sub-pixel accuracy, the accuracy of the true wavelength/wavenumber shift values associated with these peaks is rarely discussed. For the case of wavelength calibration, the wavelength of the peaks in atomic emission spectra is subject to change depending on environmental conditions; spectral line positions for the reference lamp should be corrected to account for the refractive index of air.[26,27]

In the comparison of direct vs indirect wavenumber calibration one might argue the robustness of the direct approach to environmental state. While wavelengths can be influenced by the refractive index of the observing medium (usually air or vacuum), which is subject to environmental variations, wavenumber shifts (or Raman shifts) are energy-based measurements independent of medium effect. Therefore, when using direct wavenumber calibration we do not need to take into account the environmental conditions at the specific time of recording the wavenumber reference spectrum, whereas when performing indirect calibration we must do so for the atomic emission spectrum. However, if the environmental conditions were to change (typically in the form of temperature change) following the calibration, we can no longer expect the calibration to remain accurate. Thermal expansion of the optical components, and changes in the refractive index, will induce a constant shift in the wavelength axis of the spectrometer, which will approximately cause a linear shift in the wavenumber axis. This effect can also be caused by a drift in the laser wavelength. To account for this, and to compensate by shifting back to the calibrated wavenumber shift a single wavenumber shift can be recorded from a sample such as Si.

However, a contrary argument to the superiority of wavenumber references is that they are not truly robust to temperature. The spectral lines in a Raman spectrum can change as a function of temperature, both in terms of their shape, and in terms of their Raman shift. It is known, for example, that the peaks in the cyclohexane spectrum, a commonly employed wavenumber reference standard, will change both in terms of area and offset.[28] Furthermore, the change in each peak is different and depends on the nature of the vibrational/rotational mode and also on the level of depolarisation associated with that peak; some peaks were found to vary by 25 times more than others.[28] It is difficult to account for such changes in a wavenumber reference spectrum; however, as noted in Ref. 28, for the case of cyclohexane, the temperature dependence of the Raman band position is small enough to be ignored for the calibration requirements of most applications (for example, the 802 cm^{-1} band shifts 0.12 cm^{-1} for a 10 degree C temperature change). Given the consistency of measurement in the literature to date, it can be expected that other wavenumber standards will have similar behavior. It is highly likely that the standard deviation in th Raman shift of the lines of wavenumber standards could be significantly reduced if they were always measured at a constant temperature. Indeed, there is a strong argument for always performing Raman recording in a controlled environment.

One final point of note on polynomial fitting, is the location of the outermost peaks in the reference spectrum. It is very important that peaks exists close the the ends of the recorded band; this is necessary to avoid high levels of error with polynomial fitting in bands outside of the range of the reference peaks. This has necessitated the combined use of several wavenumber reference standards in order to fully cover the bandwidth of the spectrometer. In the main paper, we develop a calibration method that is significantly more robust to this problem than polynomial fitting.

Any discussion of calibration accuracy of Raman instruments should begin with mention of the European Pharmacopoeia (Ph. Eur.),[24] which defines the tolerance for the wavenumber shift in the recording of several peaks of polystyrene, paracetamol, and cyclohexane to be ± 1.5 and ± 2.0 for bench-top and handheld instruments respectively. The accuracy of the various calibration protocols that have been reported in the literature to date vary considerably, as seen in Table 1 and 2. Given the different metrics for 'accuracy' and 'precision' that have been applied to date, it can be difficult to directly compare many of these references. We refer the reader to the relevant section in the main paper for explicit definition of these various metrics. It is notable for many papers, only the standard deviation is reported as a measure of precision while the accuracy of the calibration with respect to the known reference peaks is not reported. The reason for this is that the wavenumber shifts of these standards were not yet defined with an agreed upon accuracy. Assuming an accurate sub-pixel interpolation method is used, four factors appear to determine the calibration accuracy: (i) the resolution of the system as evidenced by the high accuracy for FT-Raman calibration;[5,7] (ii) the availability of a high number of known reference peaks over the band of interest; (iii) the sharpness of the peaks; and (iv) the accuracy of their

’known’ wavenumber shifts. These points are emphasised by the most accurate calibration protocols reported in the literature to date in Ref. 13 and Ref. 14. In Ref. 13, the authors use 67 peaks from a combined reference standard and evaluate accuracy over several months. The standard deviation of the wavenumber shifts is reported to be in the range 0.01-0.21 cm^{-1}, which is the lowest reported to date for the specific reference standards used in the study including the values reported by ASTM-E1840.[29] The absolute error with respect to the ASTM-E1840 wavenumber shifts was zero for almost all of the peaks reported. This study highlights the importance of using a large number of peaks for calibration, but also the limitation in terms of accuracy afforded by most of the calibration standards published by ASTM-E1840. In Ref. 14, gaseous reference standards are combined, which have extremely sharp peaks and for which the wavenumber shift are known down to an accuracy in the order of 0.001cm^{-1}.[30–32] In total, 45 peaks are used and the mean absolute error is reported as 0.08-0.11 cm^{-1}. However, the authors have estimated error by taking the average of the residual error in wavenumber shift of the reference peaks and the residual pixel error subsequently converted into wavenumber error. If the authors had used only residual error in the wavenumber shift of the reference peaks to define absolute error, which is the typical approach in the literature, their reported mean absolute error would be 0.001652-0.000818 cm^{-1}, which would represent the most accurate calibration reported thus far in the literature, albeit for a single spectrum.

In the main paper, we propose a wavenumber calibration protocol that advances on the background material reviewed in this section. The proposed method cannot strictly be described as a direct wavenumber calibration method since it makes use of the laser wavelength. However, it employs Raman wavenumber reference materials in the same sense as direct wavenumber calibration protocols and requires only an estimate of the laser wavelength; for this reason, we believe that the algorithm is more similar to direct wavenumber calibration than wavenumber conversion methods, which typically use wavelength reference standards and require accurate measurement of the laser wavelength. More specifically, we propose an alternative to the third-order polynomial fitting step used in existing direct wavenumber calibration protocols; although the accuracy and precision that have been reported by the state-of-the-art[13,14] is very high, we propose to augment these protocols such that they are more accurate in bands outside of the outermost peaks; we note that all of the literature to date does not report the accuracy in these bands. The method proposed here may also enable few peaks to be used and therefore reduce the number of materials used in the composite reference standard. We will demonstrate that our method has slightly higher accuracy than second and third-order fitting inside the band of the reference peaks, and significantly higher accuracy outside of this band. The proposed method is based on using a physical model of the Raman spectrometer to derive a relationship between wavenumber shift and detector pixel. The use of a physical model of the spectrometer has previously been used in Raman wavenumber calibration;[10] however, in that case the author relates wavelength to pixel and the method can therefore not be classified as a direct wavenumber protocol. Furthermore, the author defines the wavelength/pixel relationship to be governed by a second order polynomial, the coefficients of which are defined by the parameters of the optical system. This is done only once and these parameters are fixed for future re-calibration. In the main paper, we do not impose such a limitation and we assume a general rotating grating, which can be transmitting or reflecting and which requires frequent calibration.

More details on Table 1 and 2

Footnotes for Table 1 and 2 are provided here: †1: The laser wavelength is estimated using the wavenumber shift of the cyclohexane and toluene peaks. The first number in column 2 relates to peaks from the atomic emission lamp, the second number relates to combined cyclohexane and toluene peaks used to estimate laser wavelength; †2: The values are calculated from Ref3 Table vii; †3 Different first order polynomials were used to connect each set of adjacent reference peaks; †4: Polynomial fitting is not used; A physical model of the system is developed relating wavelength to pixel position, which is used to determine the wavelength axis; †5: The laser wavelength is estimated by measuring the wavenumber shift associated with the rotational lines of hydrogen; †6 18 neon peaks were used for wavelength calibration, and the laser wavelength is estimated using the wavenumber shift of 11 cyclohexane peaks. The SD values relate to a single peak of cyclohexane. The smaller value is the result of several hundred separate calibrations averaged together where the grating is slightly moved between calibrations. †7: SD values are provided as follows: indene fingerprint region SD:0.4-1 and CH-band SD:0.5-2. The MAE results given in the table relate to a simulation of calibration taken from Table I and Table II in Ref 12 using polynomials of orders 1, 2, and 3. †8: Results for Bis(MSB) and Naphthalene are compared with ASTM E1840-96.[29] Mean wavenumber shift positions over 60 measurements are identical for all peaks except four, each of which had a difference of 0.1 cm^{-1}; SD for Bis peaks are 0.02-0.21 and for Naphthalene are 0.01-0.13. †9: The maximal error of residuals in a single (combined) spectrum, 3-sigma, is actually reported as 0.24, and 0.34 for parallel and perpendicular polarisation, respectively. The authors have estimated error by taking the average of the residual error in the wavenumber shift of the reference peaks after third order polynomial fitting (this is the standard approach by other authors) and the residual pixel error, which is subsequently converted into wavenumber shift error. The latter is much larger than the former; if the authors had used the typical approach their reported errors would be MAE:0.001652 and 0.000818, which would represent the most accurate calibration reported thus far in the literature. †10: SD of 15 peaks from 4-acetamidophenol over 100 spectra ranged from 0.0374-0.5333; SD of 15 polymer peaks over 100 spectra ranged from 0.0769-0.6542. †11: Three devices, and three polynomial orders (2,3,4) were evaluated: Device 1:(3.56,3.49,3.47); device 2(0.98,0.92,0.91); device 3(0.76,0.60,0.52). †12: For three chemicals only a single peak was analysed. These are 1001.3cm^{-1} for polystyrene, 1001.1cm^{-1} for benzonitrile, 802.0cm^{-1} for cyclohexane; †13: The normalised interquartile range (nIQR) is given as an estimate of the standard deviation; †14:

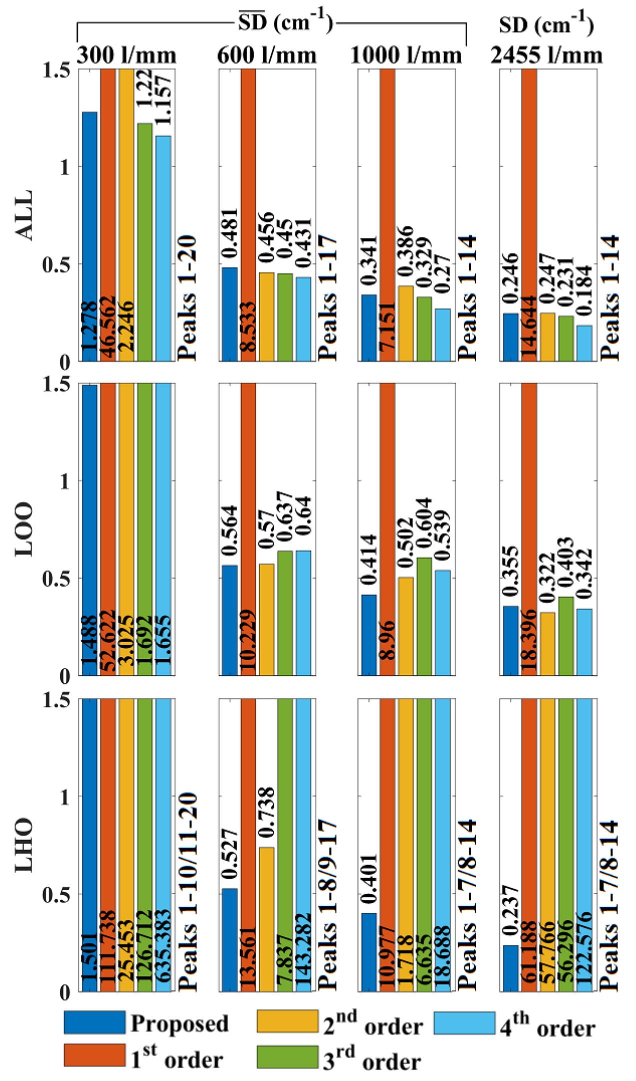

Figure 1. Wavenumber shift errors for 4-acetamidophenol using the standard deviation. The results of the algorithm proposed in this paper is given in blue and the results for first-, second-, third, and fourth-order polynomial fitting are given in orange, yellow, green, and blue, respectively.

Results are reported separately for a single spectrum of the three standards: paracetamol(MAE:0.45,SD:0.24); cyclohexane(MAE:1.93,sd2.16); polystyrene(MAE:1.31,sd 1.52).

Results using Standard Deviation and RMSE

In this appendix ALL, LOO, and LHO evaluation of the proposed algorithm is repeated for the underlying metrics of standard deviation and root mean square error, which are sometimes preferred in the literature. The definitions of these metrics are given in the main body of the paper. Evaluation using the error metric of standard deviation is given in Fig. 1, Fig. 3, Fig. 5 and using Root Mean Square Error is given in Fig. 2, Fig. 4, Fig. 6. These results correspond to those shown in Fig. 4, Fig. 5, and Fig. 6 in the main body of the paper for the case of the error metric Mean Absolute Error. These additional results are shown here to help in comparing with results from other papers.

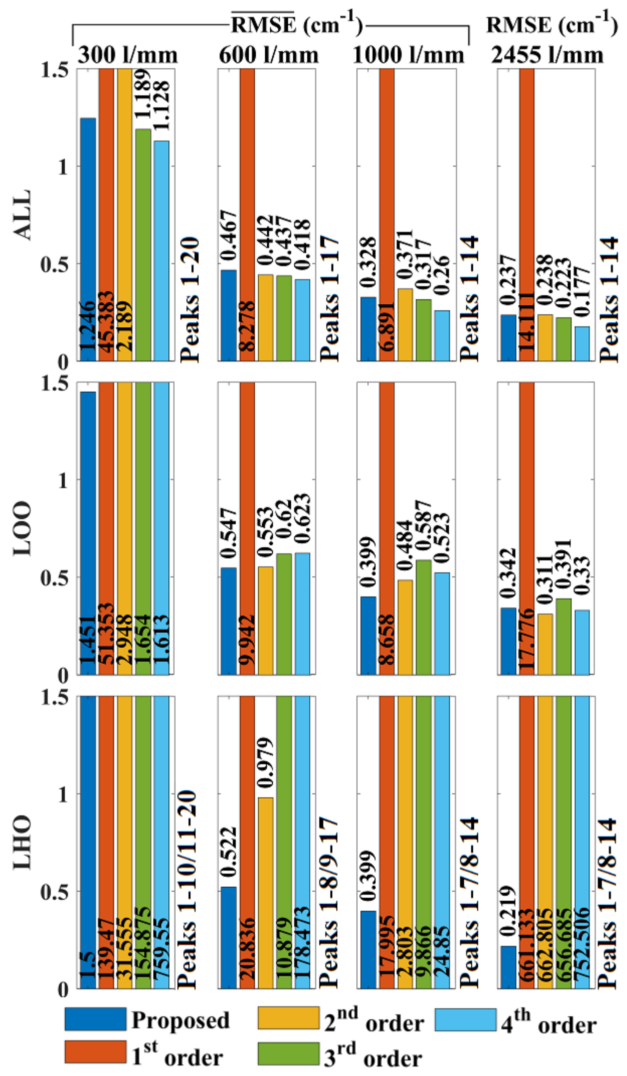

Figure 2. Wavenumber shift errors for 4-acetamidophenol using the RMSE. The results of the algorithm proposed in this paper is given in blue and the results for first-, second-, third, and fourth-order polynomial fitting are given in orange, yellow, green, and blue, respectively.

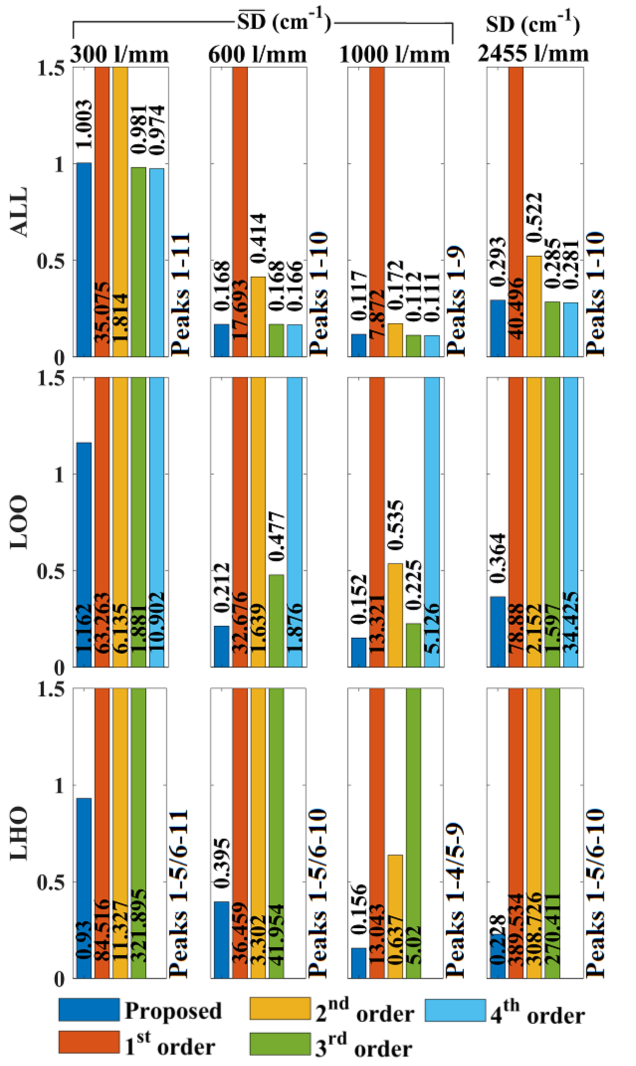

Figure 3. Wavenumber shift errors for benzonitrile using the standard deviation. The results of the algorithm proposed in this paper is given in blue and the results for first-, second-, third, and fourth-order polynomial fitting are given in orange, yellow, green, and blue, respectively.

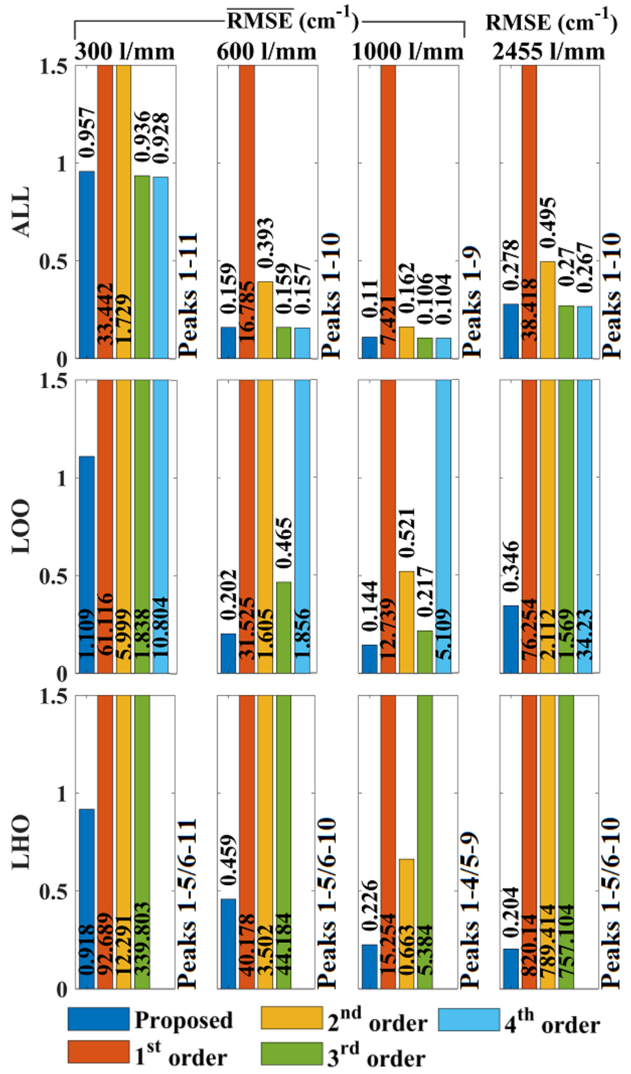

Figure 4. Wavenumber shift errors for benzonitrile using the RMSE. The results of the algorithm proposed in this paper is given in blue and the results for first-, second-, third, and fourth-order polynomial fitting are given in orange, yellow, green, and blue, respectively.

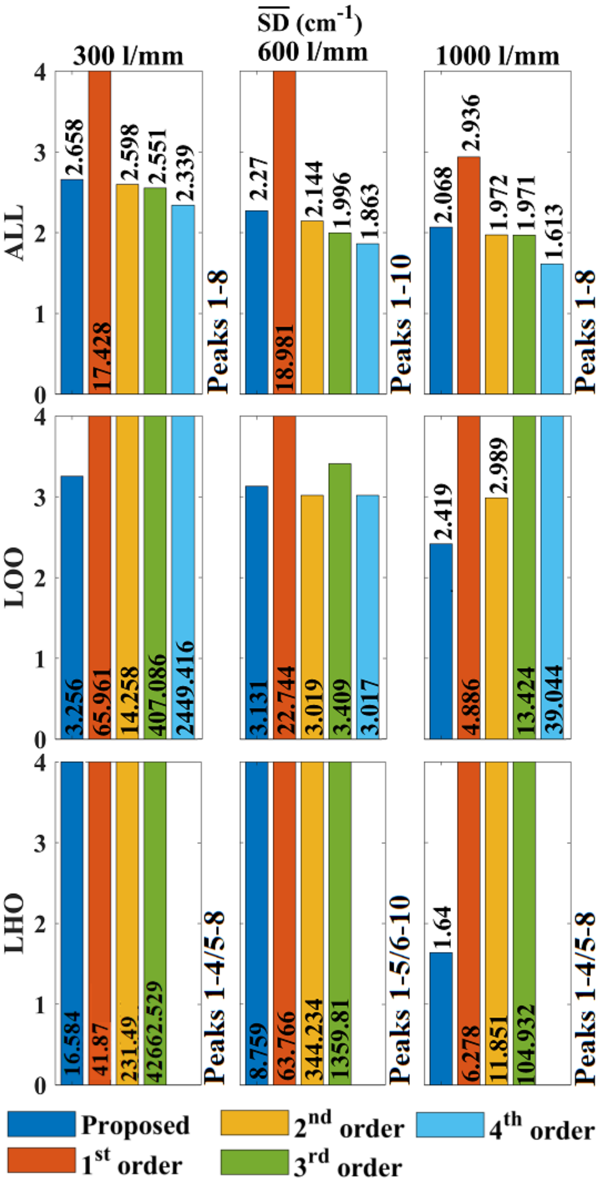

Figure 5. Wavenumber shift errors for commercial polymer using the standard deviation. The results of the algorithm proposed in this paper is given in blue and the results for first-, second-, third, and fourth-order polynomial fitting are given in orange, yellow, green, and blue, respectively.

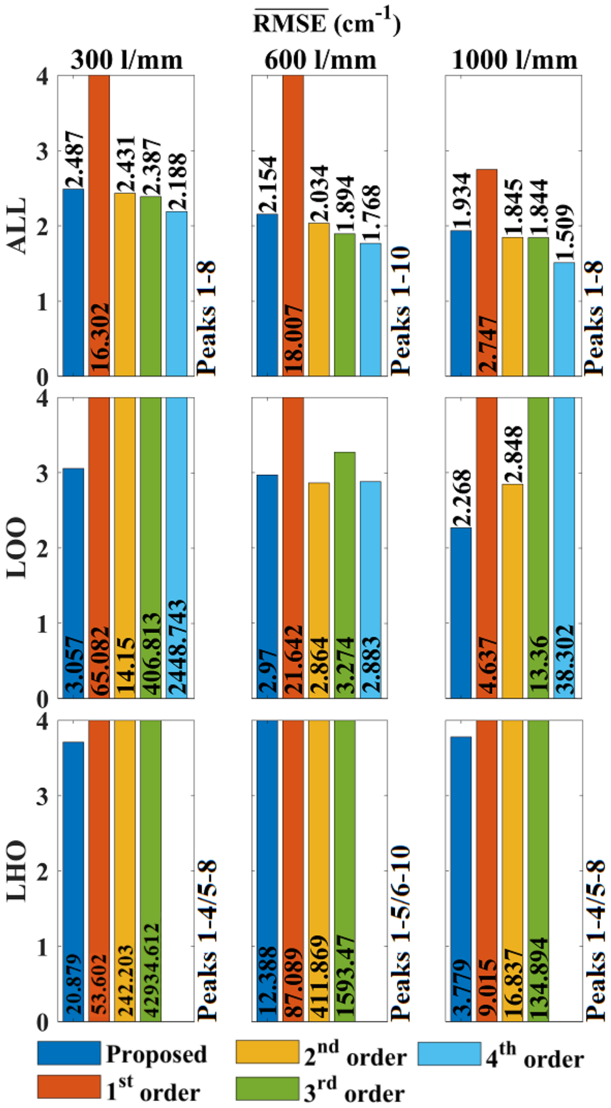

Figure 6. Wavenumber shift errors for commercial polymer using the RMSE. The results of the algorithm proposed in this paper is given in blue and the results for first-, second-, third, and fourth-order polynomial fitting are given in orange, yellow, green, and blue, respectively.

Detailed analysis of peak error

In this section, we examine the wavenumber shift error for each individual peak across the three reference spectra using each of the different metrics: ALL, LOO, and LHO. The goal is to investigate patterns in the wavenumber shift error when using the different calibration methods. More explicitly, we calculate the peak mean absolute error (PMAE) for each reference line v_i in the reference spectrum, which is defined as follows:

$$\overline{PMAE(v_i)} = \frac{1}{K}\sum_{k=1}^{K}|error_k(v_i)| \tag{1}$$

where $error_k(v_i)$ is given by the function $error(v_i)$ for the kth spectrum as defined in Equation 10 in the main text of the paper, and again K is the number of reference spectra that have been recorded, which is $K = 100$ for the experiments in this paper. The $PMAE$ function is calculated for all M spectral lines in the reference spectrum. It should be noted that the MAE function defined in Equation 2 can be rewritten in terms of this peak error function as follows:

$$\overline{MAE} = \frac{1}{M}\sum_{i=1}^{M}PMAE(v_i) \tag{2}$$

We begin in Fig. 7 by calculating the PMAE function for the wavenumber reference 4-acetamidophenol spectra that have been wavenumber calibrated using 2nd order polynomial fitting, 3rd order polynomial fitting, and using the method proposed in this paper. It should be noted that these error functions have been calculated over a dataset of $K = 100$ different reference spectra that have been recorded with movements of the grating angle. In Fig. 7 (a1) the PMAE is shown for the 20 reference peaks in 4-acetamidophenol for the case of using ALL peaks and for the case of using LOO analysis. For ALL peaks, all 20 peaks were used in the calibration routine, and then the $error_k(v_i)$ function for each v_i was measured for the calibrated axis. Therefore, for ALL peaks the calibration routine is applied once for each of the $K = 100$ spectra. As described in the paper, for LOO the $error_k(v_i)$ function is calculated on a reference line v_i, that has not been included in the calibration protocol; therefore, for a given spectrum, k, each calibration protocol is applied 20 times, using a different set of 19 peaks in each instance, and the missing wavenumber shift v_i is used to calculate the peak error defined by Equation 10 in the main body. This is repeated for each of the 100 spectra in the dataset to obtain the PMAE as defined by Equation 1; therefore, each calibration routine is applied 2000 times for LOO analysis. Results are shown for 2nd and 3rd order fitting as well as for the proposed calibration method in Fig. 7 (a1). For each of the three calibration protocols it can be seen that LOO is slightly less accurate that the ALL peaks case, which is to be expected since LOO removes the possibility of overfitting and provides a more reliable evaluation of how a calibration protocol will perform at wavenumber shift values that are not included in the discrete set of reference wavenumber lines. It is interesting to note the similar pattern of relative error across the different lines in the spectrum, particularly for 3rd order fitting and for the proposed algorithm. It is also interesting to note that for both calibration protocols the highest errors occur for the peaks at 213 cm^{-1} and 1515 cm^{-1}, which are amongst the broadest and weakest lines in the spectrum; sharper peaks have lower error, which may point to a difficulty in accurately determining sub-pixel position for broader peaks in general. Notably, the peak at 213 cm^{-1} also has one of the highest uncertainties of all of the peaks (see Table 4 in the main text). Inspection of the standard deviation values in the table reveals that those peaks with the smallest deviations appear to have the smallest errors in the figure. In Fig. 7 (a2) and (a3) the results of LHO analysis are presented. In Fig. 7 (a2) shows the error for the left 10 peaks when calibration has been applied using only the right 10 peaks. Surprisingly, third order fitting performs well in this instance; however, this appears to be a fortuitous result and when we tested again using the right 9 most peaks for calibration significant error appeared for the left 11 peaks. In Fig. 7 (a3) the error for the right most 10 peaks is shown when calibration used only the left 10 peaks; in this case only the proposed method performs well. The disparity in error in Fig. 7 (a2) and (a3) relates to the range and distribution of the 10 peaks used for calibration. The same set of results are shown for the 600 lines/mm grating in Fig. 7 (b1-b3). Once again the proposed method is the most consistent overall. Interestingly, in this case 3rd fitting performs poorly for LHO analysis for both sides, while 2nd order fitting performs better. Similar results are shown for the 1000 lines/mm grating in Fig. 7 (c1-c3) and for the 2455 grating in Fig. 7 (d1-d3); in the later case only a single spectrum is used in the dataset, i.e. $K = 1$. It is notable that the same approximate pattern of error is found for the different gratings in terms of the relative error of each peak in the 4-acetamidophenol spectrum.

A similar set of results are shown in Fig. 8 for the benzonitrile reference material for the 300 lines/mm grating in (a1-a3), 600 lines/mm grating (b1-b3), 1000 lines/mm grating in (c1-c3) and the 2455 lines/mm grating in (d1-d3). As for the previous case LOO is slightly worse than ALL for all methods and the proposed method shows slightly better error performance when compared with third order fitting for both ALL and LOO for all four gratings. However, the proposed method is clearly superior for LHO analysis is all cases.

A third set of results are shown in Fig. 9 for the polymer reference material for the 300 lines/mm grating in (a1-a3), 600 lines/mm grating (b1-b3), and the 1000 lines/mm grating in (c1-c3). For the case of the 300 lines/mm grating not all of the lines in Table 4 in the main text could be used due to the relatively low resolution of that system causing neighbouring peaks to blur and merge. For this reason, the two lines at 743 cm^{-1} and 2914 cm^{-1} were omitted for this reason. For the other two gratings all peaks listed in the table that were within the bandwidth of the spectrometer were included in the calibration and error analysis. There are a number of interesting points in relation to the results. Firstly, the peak at 1005.7 cm^{-1} consistently has the worst for each of the ALL and LOO analyses in Fig. 9 (a1), (b1) and (c1); this is in spite of the fact that this peak is reported to have the lowest uncertainty of all of the peaks (see Table 4 in the main text; this indicates that there may be a problem with the values reported by Ref. 15. The second point of interest is the high error for the right most peak at 2869 cm^{-1} in the LOO

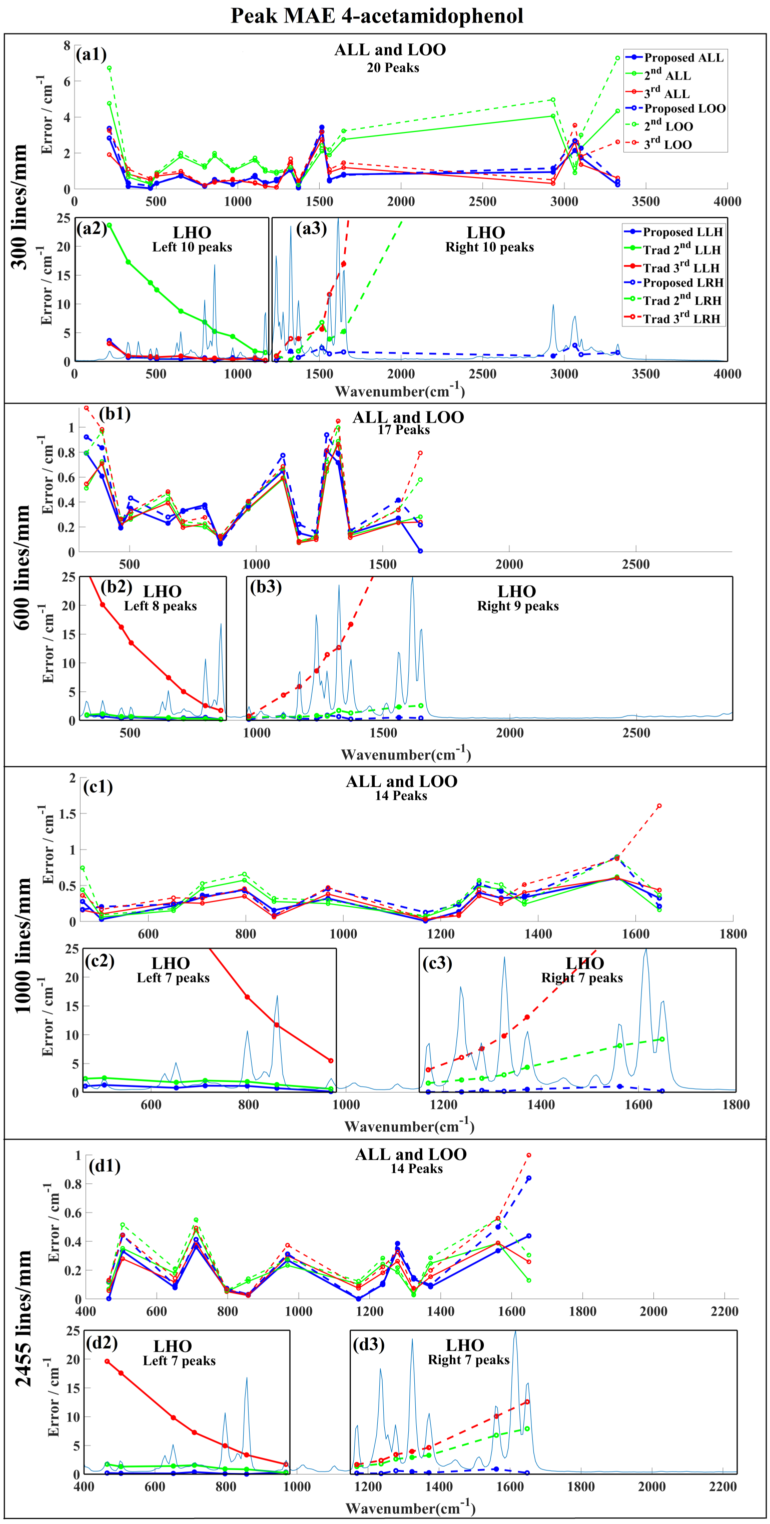

Figure 7. Peak Mean Absolute Error (PMAE) calculated for 4-acetamidophenol spectra that have been wavenumber calibrated using 2nd order polynomial fitting, 3rd order polynomial fitting, and using the method proposed in this paper. It should be noted that these error functions have been calculated over a dataset of 100 different reference spectra that have been recorded with movements of the grating angle. Results for ALL, LOO and LHO analysis are shown in (a1-a3) for the 300 lines/mm grating; in (b1-b3) for the 600 lines/mm grating; in (c1-c3) for the 1000 lines/mm grating; and in (d1-d3) for the 2455 lines/mm grating. For the latter case, the dataset contains only a single spectrum since the grating could not be rotated. See text for more details.

analysis in Fig. 9 (a1) and the high error of two right most peaks at 2869 cm^{-1} and 2914 cm^{-1} in the LOO analysis in Fig. 9 (b1). These high errors result from the long distance between these right most peaks and the other peaks, which appear in the fingerprint region. Such a long distance clearly has a deleterious effect on the results for 2nd and 3rd order fitting.

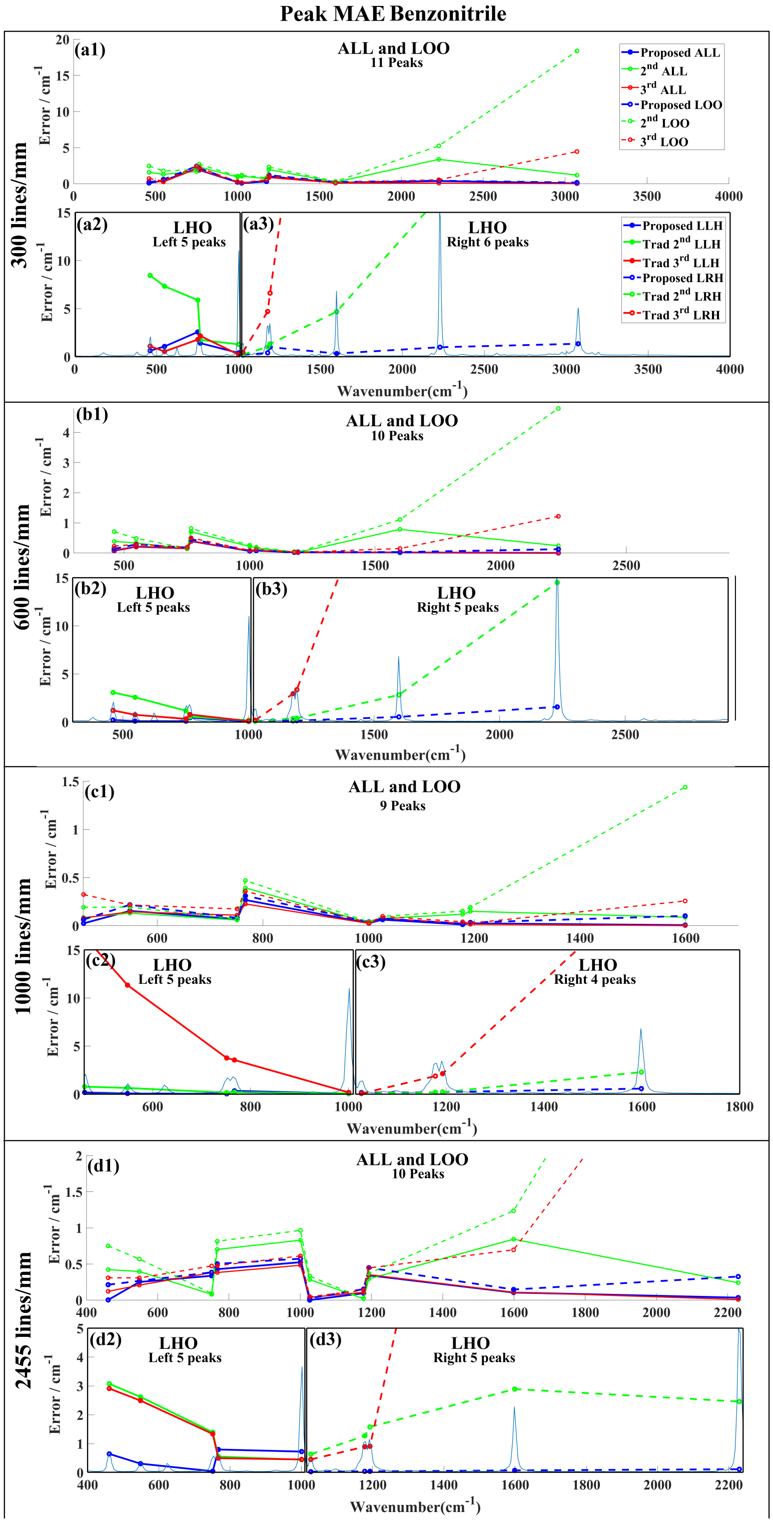

Figure 8. Peak Mean Absolute Error (PMAE) calculated for benzonitrile spectra that have been wavenumber calibrated using 2nd order polynomial fitting, 3rd order polynomial fitting, and using the method proposed in this paper. It should be noted that these error functions have been calculated over a dataset of 100 different reference spectra that have been recorded with movements of the grating angle. Results for ALL, LOO and LHO analysis are shown in (a1-a3) for the 300 lines/mm grating; in (b1-b3) for the 600 lines/mm grating; in (c1-c3) for the 1000 lines/mm grating; and in (d1-d3) for the 2455 lines/mm grating. For the latter case, the dataset contains only a single spectrum since the grating could not be rotated. See text for more details.

Effect of material dispersion on volume-phase holographic trans- mission gratings

For the volume phase transmission grating, diffraction occurs at the diffraction grating formed inside the dichromated gelatin film (with a refractive index that is much larger wavelength dispersion than that of air). Therefore, for the volume phase grating, the change in the medium refractive index with wavelength results in a change of the apparent grating period from d to $dq(\lambda)/q(\lambda_0)$ where $q(\lambda)$ is the refractive index of the film as a function of wavelength and

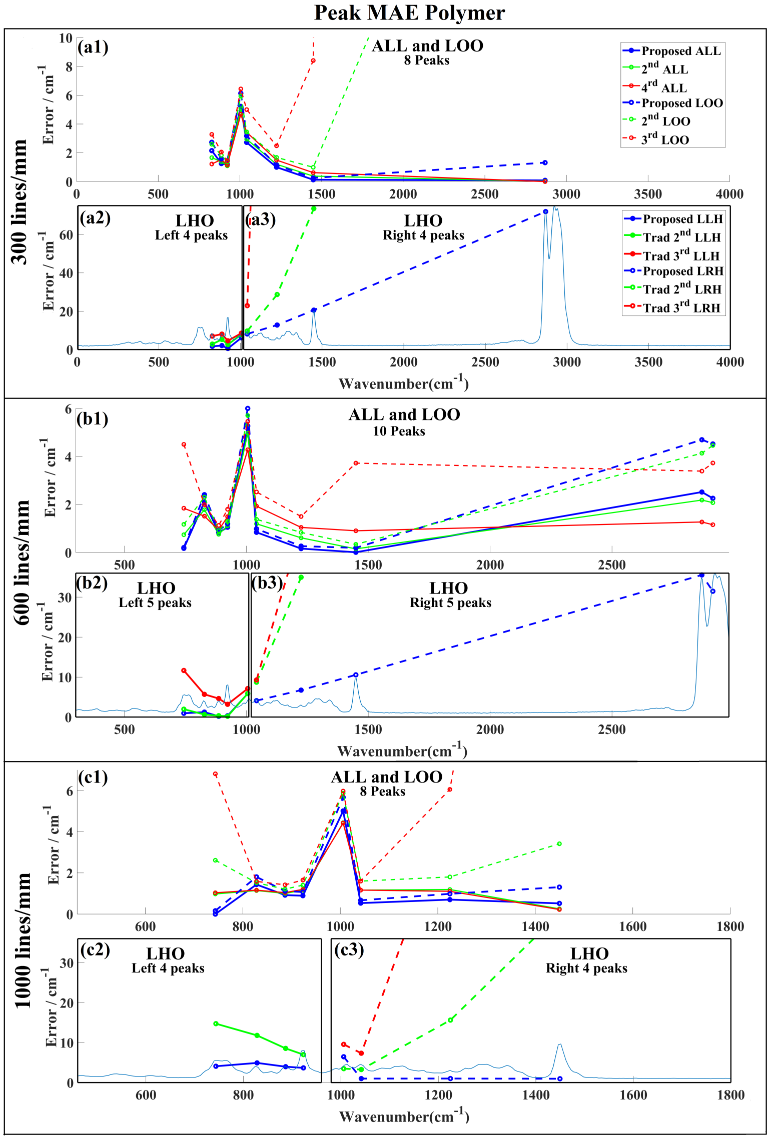

Figure 9. Peak Mean Absolute Error (PMAE) calculated for the polymer spectra that have been wavenumber calibrated using 2nd order polynomial fitting, 3rd order polynomial fitting, and using the method proposed in this paper. It should be noted that these error functions have been calculated over a dataset of 100 different reference spectra that have been recorded with movements of the grating angle. Results for ALL, LOO and LHO analysis are shown in (a1-a3) for the 300 lines/mm grating; in (b1-b3) for the 600 lines/mm grating; in (c1-c3) for the 1000 lines/mm grating; See text for more details.

λ_0 is the wavelength that is incident on the centre of the detector. This difference between the true physical model and the one used as the basis for the algorithm proposed in this paper, will affect the wavenumber calibration accuracy of the algorithm. The purpose of this section is to investigate this error further.

Including the effect of this dispersion within the dichromated gelatin film requires Equation 3 in the main text to be redefined as follows:

$$x = \frac{f}{T}\tan\left\{\theta_d + \sin^{-1}\left[\frac{n\lambda_L}{Q(v)d'(1-10^2\nu\lambda_L)} - k\sin\left(-\alpha-\theta_d\right)\right] - \alpha\right\} + \frac{C}{T} \tag{3}$$

$$Q(v) = \frac{q\left(\frac{1}{1/\lambda_L - v\times 10^2}\right)}{q\left(\frac{1}{1/\lambda_L - v_0\times 10^2}\right)}$$

where v_0 is the wavenumber shift value that is at the centre of the detector and corresponds to λ_0 in terms of wavenumber conversion. Similarly, Equation 4 must also be rewritten:

$$(v - \frac{10^{-2}}{\lambda_L})Q(v) = \frac{-10^{-2}n}{d'\left\{\sin\left[\tan^{-1}\left(\frac{xT-C}{f}\right)+\alpha-\theta_d\right]+k\sin(-\alpha-\theta_d)\right\}} \tag{4}$$

The relationship between x and v has been complicated by the presence of the nonlinear factor $Q(v)$ and the proposed algorithm can no longer be used to search for the set of parameters that fit this model. Since the algorithm cannot be adapted in a straightforward manner to account for this dispersion, we seek here to understand the error that can be expected from our algorithm in the presence of

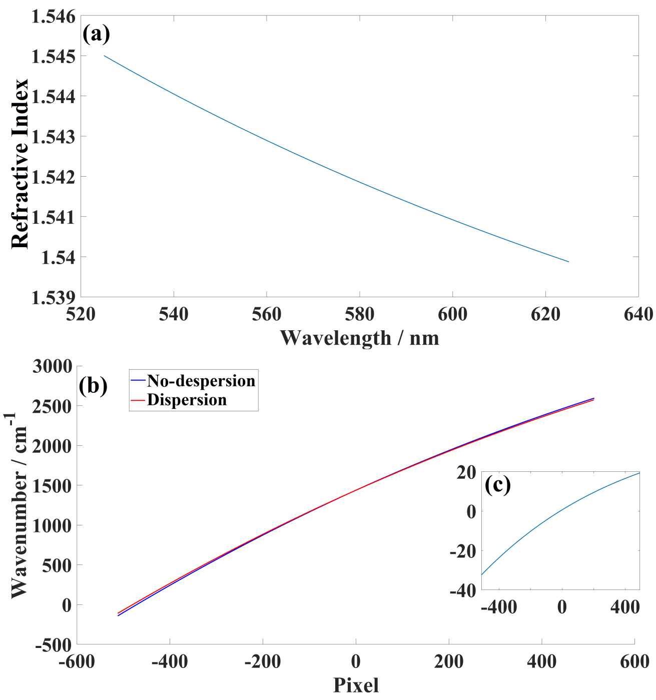

Figure 10. (a) The refractive index of gelatin over the bandwidth of the spectrometer. (b) The relationship between camera pixel and wavenumber shift that our model predicts for the set of parameters given in Table 2 where the refractive index of the gelatin film is assumed constant (blue) and where the dispersion of the gelatin film is included (red) (c) The difference in the wavenumber shift axis predicted by the two models.

such dispersion. The refractive index of gelatin at three different wavelengths ($n = 1.535$ at 833 nm, $n = 1.541$ at 633 nm, $n = 1.552$ at 486 nm) has been provided in Ref. 33. Assuming the profile of dichromated gelatin to be similar to that of gelatin and based on these three values, the coefficients a, b, and c of Cauchy's dispersion formula, $q(\lambda) = a + b/\lambda^2 + c/\lambda^4$, can be obtained by least square fitting, yielding $a = 1.5276$, $b = 4788.5$, and $c = 2.289e8$. This function is displayed in Fig. 10 (a). Making use of Equation 3 in the main text and Equation **??** as well as the parameters given in Table 2 for the transmission spectrograph, the relationship between camera pixel and wavenumber shift is plotted with and without the dispersion caused by the gelatin film in Fig. 10 (b). It can be seen that the error between the two models increases with distance from the centre pixel as shown in Fig. 10 (c).

In order to estimate the error in the wavenumber calibration provided by our algorithm, for which the model cannot account for the dispersion caused by the variation of refractive index of the gelatin film, a sequence of 20 uniformly interspersed (x, v) pairs were taken from the two plots shown in Fig. 10 (b) and fed into the algorithm, which also used the values in Table 2 as the starting parameters. The wavenumber shift errors for the resulting calibrations are shown in Fig. 11. For the case of no dispersion the MAE is $2.5E - 12$ cm^{-1} and for the case where dispersion is present, the MAE is given by 0.33 cm^{-1} with the value increasing with distance from the centre pixel. It is notable that this value is slightly higher than the experimental error (0.33 cm^{-1} for all peaks and 0.304 cm^{-1}) measured for the

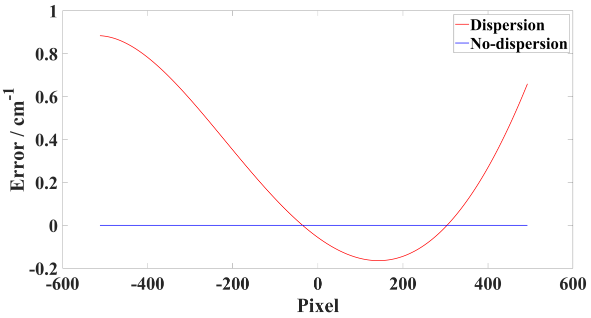

Figure 11. The calibration error of our algorithm when there exists dispersion caused by the variable refractive index of the dichromated gelatin film in the volume phase transmission grating.

Holospec spectrograph shown in Table 2 using the present method; this may indicate that the true error from dispersion is smaller than the error modelled here.

An advantage of the standard polynomial fitting approach of wavenumber calibration is that no consideration is needed about the type of the grating employed and dispersion of the grating medium and the analysis is therefore, simpler in the presence of dispersion. It is notable, however, that even though we do not attempt to account for dispersion of the film, the proposed algorithm has comparable performance to polynomial fitting using the LOO evaluation and significantly superior performance for the LHO metric. Further work is required in order to account for the effect of dispersion in the algorithm, and potentially improve these results further. For measurements on transmission spectrographs that require accuracy better than 0.33 cm^{-1} in the MAE, the present scheme will yield larger error due to neglect of wavelength of dependence of refractive index of the grating medium.

References

[1] Hennelly B and Liu D. Improved wavelength calibration by modelling the spectrometer. *Applied Spectroscopy* 2022; : 00037028221111796.

[2] Hamaguchi HO. Calibrating multichannel raman spectrometers. *Applied Spectroscopy Reviews* 1988; 24(1-2): 137–174.

[3] Vickers TJ, Rosen CA and Mann CK. Compact raman spectrometers: data handling methods. *Applied spectroscopy* 1996; 50(8): 1074–1081.

[4] Vickers TJ and Mann CK. Raman shift calibration of a compact multichannel spectrometer. *Applied Spectroscopy* 1999; 53(12): 1617–1622.

[5] Hawkins R, Hoke M and Shaw J. Wavenumber calibration of fourier transform spectra. *Applied Spectroscopy* 1983; 37(2): 134–139.

[6] Vickers TJ and Mann CK. On-line monitoring by raman spectroscopy: instrument control and calibration. In *Optical Sensors for Environmental and Chemical Process Monitoring*, volume 2367.

International Society for Optics and Photonics, pp. 219–227.

[7] Fountain III AW, Mann CK and Vickers TJ. Routine wavenumber calibration of an ft-raman spectrometer. *Applied spectroscopy* 1995; 49(7): 1048–1053.

[8] Berg RW and Nørbygaard T. Wavenumber calibration of ccd detector raman spectrometers controlled by a sinus arm drive. *Applied Spectroscopy Reviews* 2006; 41(2): 165–183.

[9] Petrov DV, Matrosov I, Sedinkin D et al. Wavenumber calibration of a multichannel raman spectrometer. In *23rd International Symposium on Atmospheric and Ocean Optics: Atmospheric Physics*, volume 10466. International Society for Optics and Photonics, p. 1046606.

[10] Holy JA. Determination of spectrometer-detector parameters from calibration spectra and the use of the parameters in spectrometer calibrations. *Applied spectroscopy* 2004; 58(10): 1219–1227.

[11] Fountain AW, Vickers TJ and Mann CK. Factors that affect the accuracy of raman shift measurements on multichannel spectrometers. *Applied spectroscopy* 1998; 52(3): 462–468.

[12] Carter DA, Thompson WR, Taylor CE et al. Frequency/wavelength calibration of multipurpose multichannel raman spectrometers. part ii: calibration fit considerations and calibration standards. *Applied spectroscopy* 1995; 49(11): 1561–1576.

[13] Hutsebaut D, Vandenabeele P and Moens L. Evaluation of an accurate calibration and spectral standardization procedure for raman spectroscopy. *Analyst* 2005; 130(8): 1204–1214.

[14] Raj A, Kato C, Witek HA et al. Toward standardization of raman spectroscopy: Accurate wavenumber and intensity calibration using rotational raman spectra of h2, hd, d2, and vibration–rotation spectrum of o2. *Journal of Raman Spectroscopy* 2020; 51(10): 2066–2082.

[15] Liu D, Byrne HJ, O'Neill L et al. Investigation of wavenumber calibration for raman spectroscopy using a polymer reference. In *Optical Sensing and Detection V*, volume 10680. SPIE, pp. 486–497.

[16] Wollman S and Bohn P. Evaluation of polynomial fitting functions for use with ccd arrays in raman spectroscopy. *Applied Spectroscopy* 1993; 47(1): 125–126.

[17] Fales AM, Ilev IK and Pfefer TJ. Evaluation of standardized performance test methods for biomedical raman spectroscopy. *Journal of Biomedical Optics* 2021; 27(7): 074705.

[18] Ryabchykov O, Guo S and Bocklitz T. Analyzing raman spectroscopic data. *Physical Sciences Reviews* 2019; 4(2).

[19] Itoh N, Shirono K and Fujimoto T. Baseline assessment for the consistency of raman shifts acquired with 26 different raman systems and necessity of the standardization calibration protocol. *Analytical Sciences* 2019; : 18P501.

[20] Guo S, Beleites C, Neugebauer U et al. Comparability of raman spectroscopic configurations: a large scale cross-laboratory study. *Analytical Chemistry* 2020; 92(24): 15745–15756.

[21] Weatherall JC, Barber J, Brauer CS et al. Adapting raman spectra from laboratory spectrometers to portable detection libraries. *Applied Spectroscopy* 2013; 67(2): 149–157.

[22] Carrabba MM. Wavenumber standards for r aman spectrometry. *Handbook of vibrational spectroscopy* 2006; .

[23] Itoh N and Shirono K. Reliable estimation of raman shift and its uncertainty for a non-doped si substrate (nmij crm 5606-a). *Journal of Raman Spectroscopy* 2020; 51(12): 2496–2504.

[24] COUNCIL OE. European pharmacopoeia. *Strasbourg, France: Council of Europe* 2016; .

[25] Shen C, Vickers TJ and Mann CK. Abscissa error detection and correction in raman spectroscopy. *Applied spectroscopy* 1992; 46(5): 772–777.

[26] Birch K and Downs M. An updated edlén equation for the refractive index of air. *Metrologia* 1993; 30(3): 155.

[27] Ciddor PE. Refractive index of air: new equations for the visible and near infrared. *Applied optics* 1996; 35(9): 1566–1573.

[28] Pelletier M. Effects of temperature on cyclohexane raman bands. *Applied spectroscopy* 1999; 53(9): 1087–1096.

[29] ASTM E1840-96(2014). Standard guide for Raman Shift standards for spectrometer calibration. Standard, ASTM International, West Conshohocken,Pennsylvania, 2014.

[30] Komasa J, Piszczatowski K, Łach G et al. Quantum electrodynamics effects in rovibrational spectra of molecular hydrogen. *Journal of chemical theory and computation* 2011; 7(10): 3105–3115.

[31] Pachucki K and Komasa J. Rovibrational levels of hd. *Physical Chemistry Chemical Physics* 2010; 12(32): 9188–9196.

[32] Edwards H, Long D, Najm K et al. The vibration-rotation raman spectra of 18o2, 17o18o, 17o2 and 16o2. *Journal of Raman Spectroscopy* 1981; 10(1): 60–63.

[33] Bonal V, Quintana JA, Villalvilla JM et al. Simultaneous determination of refractive index and thickness of submicron optical polymer films from transmission spectra. *Polymers* 2021; 13(15): 2545.
